# Recent Progress in Large‐Area Organic Solar Cells

**DOI:** 10.1002/smsc.202300004

**Published:** 2023-04-13

**Authors:** Ben Zhang, Fu Yang, Yaowen Li

**Affiliations:** ^1^ Laboratory of Advanced Optoelectronic Materials Suzhou Key Laboratory of Novel Semiconductor-optoelectronics Materials and Devices College of Chemistry, Chemical Engineering and Materials Science Soochow University Suzhou 215123 China; ^2^ Jiangsu Key Laboratory of Advanced Negative Carbon Technologies Soochow University Suzhou Jiangsu 215123 P. R. China; ^3^ State and Local Joint Engineering Laboratory for Novel Functional Polymeric Materials Jiangsu Key Laboratory of Advanced Functional Polymer Design and Application College of Chemistry, Chemical Engineering and Materials Science Soochow University Suzhou 215123 China

**Keywords:** coating techniques, flexible transparent electrodes, large-area OSCs, materials requirements, morphology optimization

## Abstract

Organic solar cells (OSCs) attract significant attention due to their great potential in flexible, lightweight, and low‐cost photovoltaic technology. Given the reformation of non‐fullerene acceptors, the certificated power conversion efficiency (PCE) of single‐junction OSCs has developed rapidly over 19% in the small device size (<1 cm^2^). However, the PCEs of large‐area OSCs are significantly lower than that of small‐area devices. Several essential issues in upscaling OSCs from small‐area to large‐area need to be overcome to bridge the efficiency gap, including coating techniques, material requirements, morphology optimization, flexible transparent electrodes development, and devices stability research. Herein, recent progress and challenges in materials and processing technologies of large‐area OSCs are summarized. Based on the analysis, strategies and opportunities are proposed to promote the development of large‐area efficient OSCs toward mass production.

## Graphical abstract

A comprehensive overview of the latest research progress of the high‐performance large‐area organic solar cells (OSCs), including coating techniques, material requirements, morphology optimization, flexible transparent electrodes, and device stability, is provided. Finally, an outlook and perspectives are provided for the future development of large‐area OSCs with respect to industrial‐scale production and application.

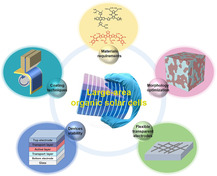

## 1 Introduction

Organic solar cells (OSCs) possess the advantages of low cost, intrinsic flexibility, and large‐area printing.^[^ 1, 2, 3, 4 ^]^ These merits promote OSCs to be widely deployed in portable energy resources and building‐integrated photovoltaics in the future.^[^ 5, 6 ^]^ Since the first report on bulk‐heterojunction (BHJ) solar cells in 1995,^[^ ^7^ ^]^ fullerene acceptors have dominated OSCs for over two decades due to their high electron affinity and mobility.^[^ 8, 9 ^]^ However, the weak absorption in the near‐infrared (NIR) region, poor energy‐level tunability, and morphological instability of the fullerene acceptors limited their further development.^[^ 10, 11 ^]^ Therefore, researchers have made tremendous efforts toward the development of non‐fullerene acceptors (NFAs) over the past several years. In 2015, Zhan et al.^[^ ^12^ ^]^ invented a high‐performance fused‐ring NFA 3,9‐bis(2‐methylene‐(3‐(1,1‐dicyanomethylene)‐indanone)‐5,5,11,11‐tetrakis(4‐hexylphenyl)‐dithieno[2,3‐d:2′,3′‐d′]‐s‐indaceno[1,2‐b:5,6‐b′]‐dithiophene (ITIC) with strong and broad absorption in the visible and NIR regions, appropriate energy level matched with low‐bandgap donor polymers, and good donor/acceptor miscibility. Since then, a series of new high‐performance NFAs have been sprung out, and the champion power conversion efficiency (PCE) of the NFA‐based OSCs has exceeded 19% in the small‐area device size (<1 cm^2^).^[^ 13, 14, 15 ^]^

**Figure 1:**
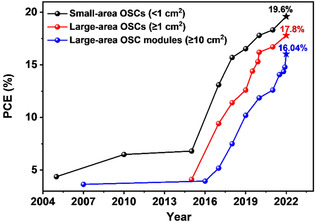
The trend of the high‐performance small‐area (<1 cm^2^), large‐area (≥1 cm^2^) organic solar cells (OSCs) and OSC modules (≥10 cm^2^).

To date, the most common solution‐based technique for the preparation of efficient OSCs is the spin‐coating method due to its simplicity and high reproducibility of film thickness and morphology in small‐area preparation.^[^ 16, 17 ^]^ However, the morphology and thickness of the active layer are determined by the centrifugal force during the spin‐coating, which cannot be realized in scalable coating processes and is incompatible with continuous production (e.g., roll‐to‐roll [R2R]).^[^ 18, 19, 20 ^]^ Therefore, alternative scalable techniques can be easily transferred R2R manufacturing need to be explored, such as blade‐coating, slot‐die coating, gravure printing, inkjet printing, and so on. Unfortunately, the large‐area OSCs and OSC modules prepared by these techniques are still lower than small‐area devices due to the difficulty in controlling the uniformity, thickness, and crystallization of the large‐area films.^[^ 18, 21, 22, 23, 24, 25 ^]^ **Figure** **1**a shows the trend of the high‐performance small‐area, large‐area OSCs and OSC modules. Improvement of photovoltaic performance of large‐area devices depends on understanding film‐formation mechanism and fluid mechanics based on different coating techniques, and developing materials for the high‐performance photoactive layer and interface layer (especially the cathode interface layer [CIL]).

In addition, large‐area OSCs fabricated on flexible substrates should also receive sufficient attention to realize the practical application of flexible and portable power sources.^[^ 26, 27 ^]^ Currently, the flexible transparent electrode (FTE) is one of the key factors affecting the photovoltaic and mechanical properties of large‐area flexible OSCs. The high‐performance FTEs should possess high optical transparency, low surface roughness, low sheet resistance, and good mechanical bending durability.^[^ 26, 28, 29, 30 ^]^ However, traditional indium tin oxide (ITO) transparent electrode is a brittle semiconductor, and its conductivity decreases with mechanical deformation, which limits its application in large‐area flexible OSCs. To date, many researchers have tried to develop ITO alternatives for realizing the application in large‐area flexible OSCs, including conductive polymers,^[^ 31, 32 ^]^ carbon‐based materials,^[^ 33, 34 ^]^ metal nanowires,^[^ 35, 36 ^]^ and metal grids.^[^ 37, 38 ^]^ Even excellent PCEs over 17% were obtained for the flexible OSCs based on silver nanowire (AgNW) flexible FTEs,^[^ ^39^ ^]^ high‐performance large‐area flexible OSCs still lag behind. Therefore, the development of high‐performance large‐area FTEs is crucial for accelerating the further application of flexible OSCs.

In this review, we provide a comprehensive overview of the latest research progress of the large‐area OSCs, mainly focusing on 1) recent progress in the development of coating techniques for large‐area OSCs; 2) organic active and interface materials for satisfying the requirements of high‐thickness insensitivity, and large‐area adaptability; 3) film‐formation mechanism in regulating the morphology of large‐area active layers; 4) that systematically evaluate the recent progress in high‐performance FTEs; and 5) that summarize devices stability improvement strategies. Finally, we provide an outlook and perspectives for the future development of large‐area OSCs with respect to industrial‐scale production and application.

## 2 Large‐Area Coating Techniques

To meet the requirements of commercialization, large‐area OSCs should be able to be manufactured through scale‐up coating techniques, especially those methods that can be transferred into R2R manufacturing.^[^ 40, 41, 42 ^]^ The high‐quality active layer is a key factor for high‐efficiency large‐area OSCs. Blade‐coating, slot‐die coating, gravure printing, and inkjet printing are the most explored techniques for preparing large‐area active layers. However, it is important to note that the fluid mechanics and crystallization during the scalable coating process differ significantly from spin coating, resulting in inferior uniformity in the large‐area film.^[^ 43, 44 ^]^ Mounting evidence in the literature demonstrates coating techniques have a crucial influence on film morphology, highlighting the significance of fine‐tuning their processing parameters. In the following, we will introduce in detail the methods of different coating techniques for mass manufacturing. The performance of OSCs devices with different coating methods mentioned in this section is shown in **Table** **1**.

**Table 1:** The performance of OSCs devices with different coating methods

Active layer	Coating method	Area [cm^2^]	*V*_oc_ [V]	*J*_sc_ [mA cm^−2^]	FF [%]	PCE [%]	References
PBDB‐T:ITIC	Blade‐coating	0.04	0.88	17.14	66.10	10.03	[45]
PBDB‐T:PTB7‐Th:FOIC	Blade‐coating	0.04	0.73	25.04	65.78	11.80	[48]
PM6:BTP‐BO‐4Cl	Blade‐coating	18.73	5.77	3.66	69.94	14.79	[49]
PM6:CH7	Blade‐coating	25.2	6.01	3.42	70.17	14.42	[24]
PM6:Y6	Blade‐coating	11.52	3.20	6.41	57.85	11.86	[21]
PM6:Y6	Slot‐die coating	0.04	0.81	26.6	70.3	15.2	[50]
D18:Y6:BTR‐Cl	Slot‐die coating	0.04	0.86	26.9	74.4	17.2	[51]
PBDB‐T:ITIC	Slot‐die coating	1.04	0.86	16.96	65.23	9.77	[52]
		15	5.10	2.79	60.61	8.90	[52]
PTB7‐TH:PC_71_BM	Gravure printing	0.09	0.75	15.74	56.00	6.61	[53]
PM6:Y6	Gravure printing	1	0.83	24.98	66.03	13.61	[54]
P3HT:PCBM	Inkjet printing	0.8	0.56	5.49	57.1	1.76	[55]
PM6:BTP‐BO‐4Cl	Inkjet printing	0.09	0.85	24.07	64.00	13.09	[56]

### 2.1 Blade‐Coating

**Figure 2:**
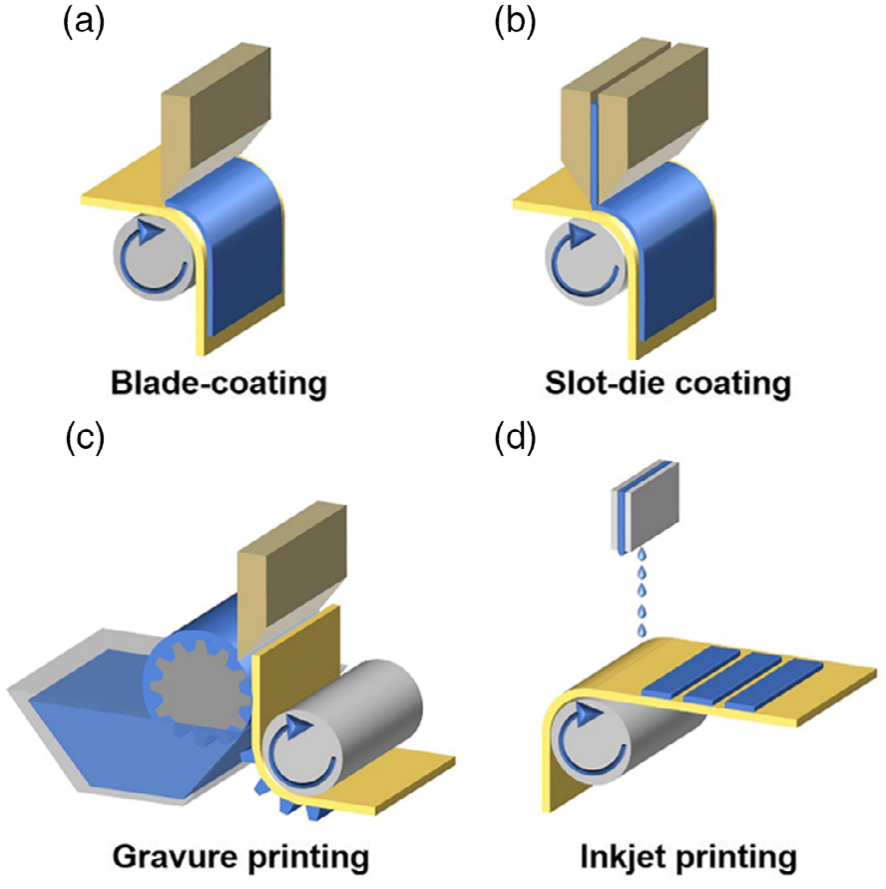
Common scalable solution deposition techniques for large‐area OSCs. a) Blade‐coating. b) Slot‐die coating. c) Gravure printing. d) Inkjet printing.

**Figure 3:**
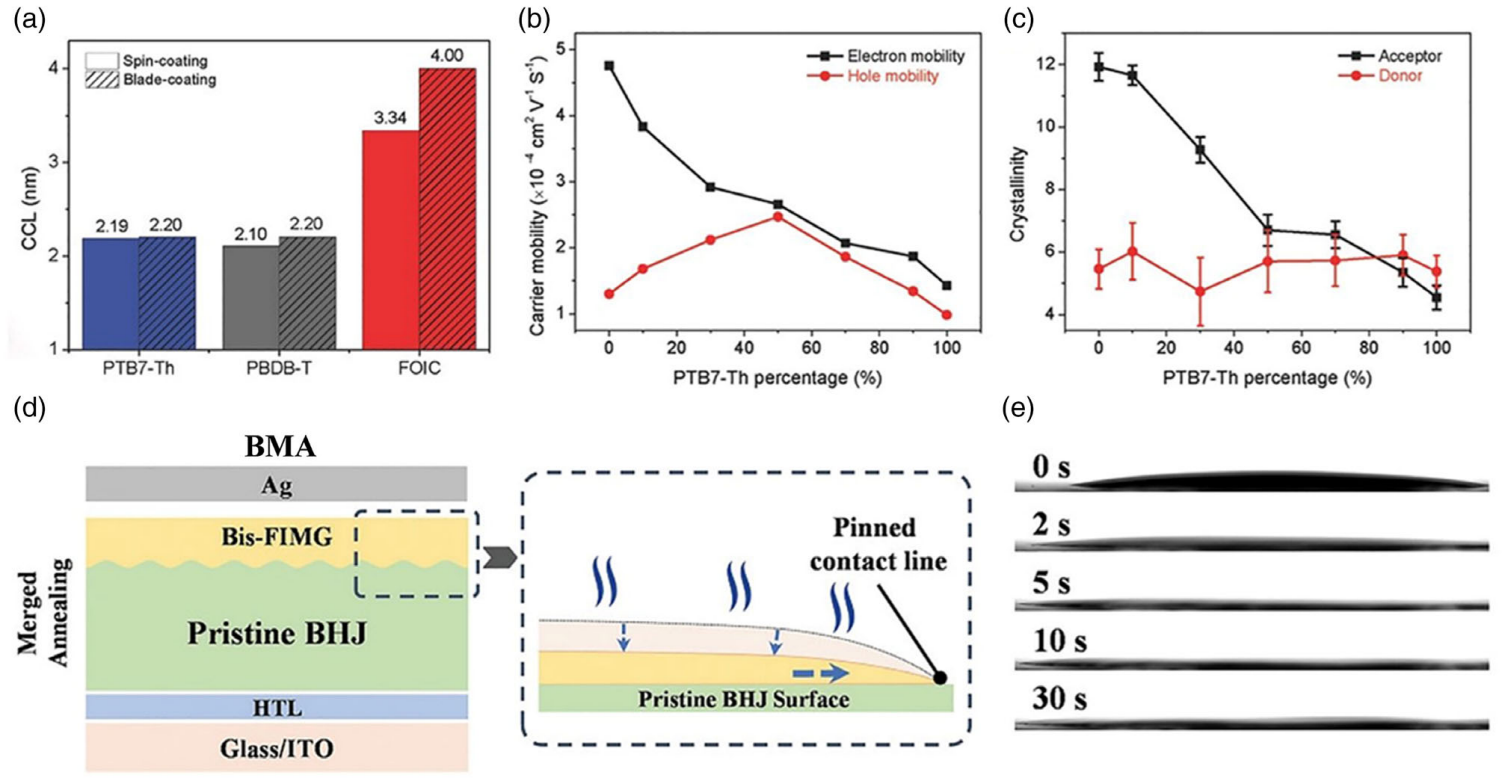
a) The calculated crystallization coherence length values of (010) peak of PBDB‐T, FOIC, and PTB7‐Th films prepared by spin‐coating and blade‐coating. b) The carrier mobility and c) the calculated crystallinity at various PTB7‐Th content. a–c) Reproduced with permission.^[^ ^48^ ^]^ Copyright 2018, Wiley‐VCH. d) Schematic illustration of device structures and drying dynamic of electron‐transport layer (ETL) meniscus via BMA processing. e) Spreading process of ETL droplets atop of the pristine BHJ films under ambient conditions. d,e) Reproduced with permission.^[^ ^49^ ^]^ Copyright 2022, Wiley‐VCH.

Blade‐coating is the most frequently explored method next to spin‐coating in lab‐scale experiments for fabricating films of well‐defined thickness (**Figure** **2**a). During the blade‐coating process, the substrate is first placed on a platform and then a blade is used to spread the organic solution on the substrate to form a wet film. Finally, as the solvent evaporates, the organic material slowly crystallizes and forms a thin film. Ma et al.^[^ ^45^ ^]^ fabricated the PBDB‐T:ITIC device by spin‐coating and blade‐coating with various 1,8‐diiodooctane (DIO) contents. The highest PCE of 10.03% can be achieved for the blade‐coated devices with 0.25% DIO additive, which is higher than the optimal spin‐coated devices with a higher DIO content of 1%. It is demonstrated that blade‐coating can induce a higher degree of molecular packing for both donors and acceptors as it helps to produce a seeding film containing numerous crystal grains that subsequently provide nucleation sites for the residual solution.^[^ 46, 47 ^]^ Subsequently, they blade‐coated ternary OSCs based on poly[2,6‐(4,8‐bis(5‐(2‐ethylhexyl)thiophen‐2‐yl)benzo[1,2‐b:4,5‐b′]dithiophene)‐*co*‐(1,3‐di(5‐thiophene‐2yl)‐5,7‐bis(2‐ethylhexyl)‐benzo[1,2‐c:4,5‐c′]dithiophene‐4,8‐dione)] (PBDB‐T):poly([2,6′‐4,8‐di(5‐ethylhexylthienyl)benzo[1,2‐b;3,3‐b]dithiophene]{3‐fluoro‐2[(2ethylhexyl)carbonyl]thieno[3,4‐b]thiophenediyl}) (PTB7‐Th):3,9‐bis(2‐methylene‐((3‐(1,1‐dicyanomethylene)‐fluoro)‐indanone))‐5,5,11,11‐tetrakis(4‐hexylphenyl)‐dithieno[2,3‐d:2′,3′‐d′]‐s‐indaceno[1,2‐b:5,6‐b′]dithiophene (FOIC) blends with different amounts of PTB7‐Th.^[^ ^48^ ^]^ They proposed that blade‐coating method can induce crystallization of all these three materials with increasing carrier mobility, but the degree of induction is different (FOIC > PBDB‐T > PTB7‐Th), resulting in an unbalanced mobility in the blade‐coated PBDB‐T:FOIC device, of which the electron mobility is much higher than the hole mobility (**Figure** **3**a–c). As a result, the optimal blade‐coated device based on PBDB‐T:PTB7‐Th:FOIC blends at PTB7‐Th content of 50% presents the highest PCE of 12.02%. Li et al.^[^ ^49^ ^]^ developed a high‐performance PM6:BTP‐BO‐4Cl‐based OSC module via a bilayer‐merged‐annealing (BMA)‐assisted blade‐coating strategy, achieving an impressive PCE of 14.79% with an active area of 18.73 cm^2^. BMA can effectively address the de‐wetting issues between polar charge‐transport layer solution and nonpolar BHJ blends, hence improving the film coverage, along with electronic and electric contacts of multi‐stacked photoactive layers (Figure 3d,e). Chen et al.^[^ ^24^ ^]^ reported an NFA CH7 with the extended conjugation central unit and long‐branched side chains. The long‐branched alkyl chains can suppress molecular excessive aggregation and ensure the good solubility of CH7 in non‐halogen solvent *o*‐xylene. As a result, the blade‐coated large‐area module with an active layer area of 25.2 cm^2^ was achieved PCE of 14.42%. Min et al.^[^ ^21^ ^]^ reported a layer‐by‐layer (LBL)‐processing approach as a printable strategy for high‐performance large‐area OSC module. The LBL method exhibits unique advantages of combining the merits of high photo‐absorption rate, suitable vertical phase separation, and good practicability, endowing the LBL devices with excellent charge‐transport and extraction properties. As a result, the LBL‐based module with an active area of 11.52 cm^2^ deliver 11.86% PCE compared with BHJ‐based 10.15%. Nevertheless, it needs to mention that there will be a thickness variation for the large‐area over 10 × 10 cm^2^ as the continuing consumption of solution during the blade‐coating process. The blade‐coating method also cannot be used in high‐throughput continuous production and is difficult to integrate into R2R equipment, but can serve as a lab‐scale analog to another meniscus‐coating method, the slot‐die coating method owing to the similar working mechanism.

### 2.2 Slot‐Die Coating

**Figure 4:**
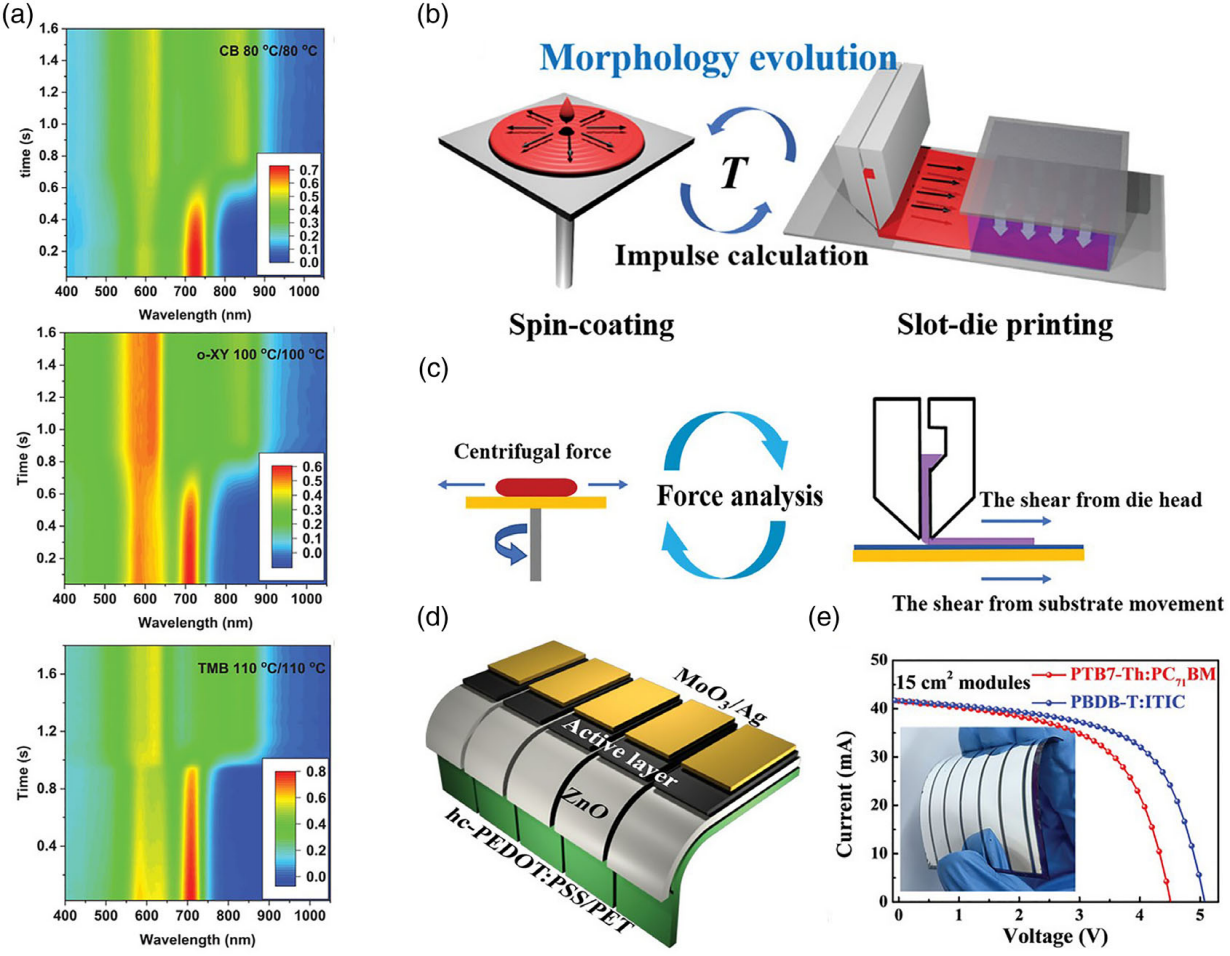
a) Time evolution of UV–vis absorption spectra during slot‐die coating at different conditions. Reproduced with permission.^[^ ^50^ ^]^ Copyright 2020, Wiley‐VCH. b) Schematic diagram of morphology evolution and c) force analysis between spin‐coating and slot‐die coating. d) The device structure of flexible OSC module. e) Current density–voltage curves of the flexible OSCs by slot‐die coating. b–e) Reproduced with permission.^[^ ^52^ ^]^ Copyright 2019, Wiley‐VCH.

The slot‐die coating technique is considered one of the most appropriate coating techniques for large‐area OSCs as it enables the deposition of either a single layer or stacks of layers one above the other with no material loss. As shown in Figure 2b, the coating solution is continuously fed into the coating head via a piston pump or a pressure tank, then is translated perpendicularly to the substrate to form the meniscus. And the thickness of the film can be precisely defined by adjusting the solution‐feeding rate, the viscosity of solution and the speed of the slot‐die head or the substrate. The evaporation rate of the solution usually affects the morphology of the coated film. Ma et al.^[^ ^50^ ^]^ demonstrated that the slot‐die coating at high‐temperature enables a higher degree of crystallinity and a smaller phase‐separation structure in the blend films, resulting in improved and more balanced carrier mobility and reduced recombination. Furthermore, they obtained similar film morphology by the different processing solvents (chlorobenzene [CB], *o*‐xylene, trimethylbenzene) during high‐temperature slot‐die‐coating method, due to the similar drying kinetics and film‐formation mechanism (**Figure** **4**a). As a result, combining a hot solution and a hot substrate, the slot‐die coated PM6:Y6‐based OSCs processed from those three solvents exhibit high comparable PCEs around 13.8%. Subsequently, Ma et al.^[^ ^51^ ^]^ showed that adding the third‐component BTR‐Cl to D18:Y6 successfully regulated the phase‐separation kinetics of the D18:Y6 film due to the better miscibility of BTR‐Cl and D18. The incorporation of BTR‐Cl tends to drive Y6 separation from the mixed amorphous D18 phase, leading to early aggregation of Y6 in the ternary blend, and improves the crystallization. Impressively, OSCs with the slot‐die coated 110 and 300 nm ternary D18:Y6:BTR‐Cl film exhibit high PCEs of 16.3% and 14.0%, respectively. Chen et al.^[^ ^52^ ^]^ demonstrated a general approach to upscale flexible OSCs to the module scale with less performance loss. They ingeniously used the shear impulse during coating/printing process to optimize the morphology evolution of both fullerenes‐based system (PTB7‐Th:PC_71_BM) and non‐fullerenes‐based system (PBDB‐T:ITIC), and detected a quantitative transformation factor of shear impulse between slot‐die printing and spin‐coating (Figure 4b,c). The evolution of the BHJ morphology at the initial step of film formation becomes reproducible if the shear impulse of each coating method is controlled. As a result, 15 cm^2^ flexible OSC modules with an efficiency up to 8.90% are successfully fabricated with excellent mechanical flexibility and operating stability (Figure 4d,e). It is worth noting that the slot‐die coating method is generally less suitable for developing expensive precursors, as it usually requires a large amount of coating solution to fill the slot head before starting the coating process.

### 2.3 Gravure Printing

**Figure 5:**
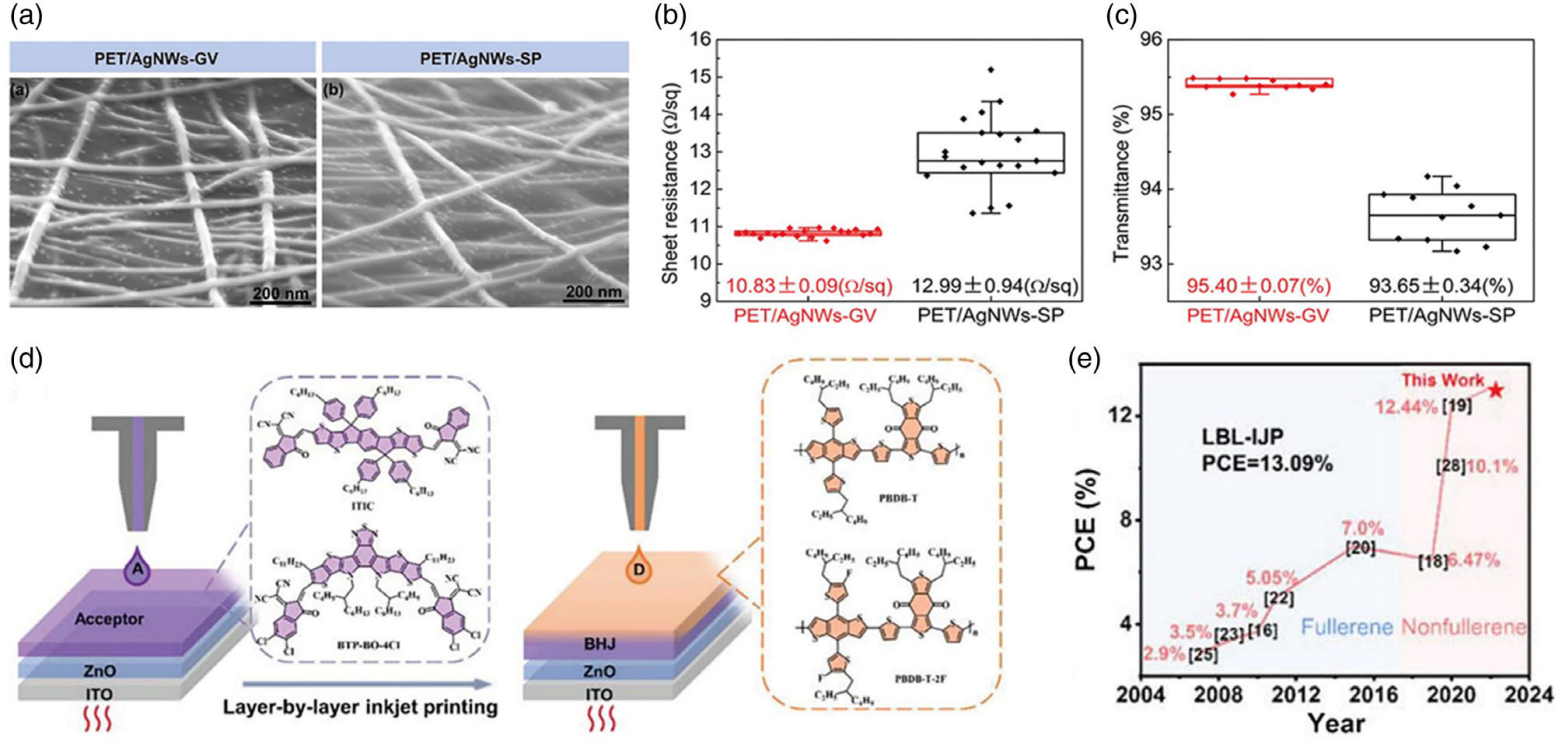
a) Scanning electron microscopy (SEM) images of the PET/AgNWs‐GV and spin‐coated AgNW (PET/AgNWs‐SP) electrodes. b) Sheet resistance and c) transmittance (at 550 nm) of the PET/AgNWs‐GV and PET/AgNWs‐SP electrodes. a–c) Reproduced with permission.^[^ ^54^ ^]^ Copyright 2020, Wiley‐VCH. d) Diagram illumination of LBL–inkjet printing process and molecular structures of donors and acceptors. e) Summary of efficiency of the inkjet‐printed OSCs in recent years. d,e) Reproduced with permission.^[^ ^56^ ^]^ Copyright 2022, Wiley‐VCH.

Gravure printing is a more complex version of knife‐overedge coating method that can be used in the high‐throughput printing of electronics (Figure 2c). It comprises a two‐roller system, the printing roller, and the supporting roller, where the printing roller has an engraved pattern, and the other roller guides the web. During the printing process, the printing roller is partially immersed in the solution container to make the engravings filled with solution. A doctor blade is used to remove the excess solution from the inactive area of the printing roller. The solution is then transferred from the printing roller to the flexible substrate through applying pressure with an embossing roller. Yang et al.^[^ ^53^ ^]^ optimized the properties of micro‐gravure R2R‐printed zinc oxide (ZnO) film through regulating the web tension, substrate pretreatment, and printing speed, and obtained high‐quality and thickness‐controllable ZnO thin film. They found that the coverage and thickness of ZnO thin film are strongly dependent on the R2R printing roller speed. The increased speed can provide more ZnO solution transferred from the printing roller onto poly(ethylene terephthalate) (PET)/ITO substrate, resulting in the enhancement of ZnO thin‐film coverage. In addition, the inverted OSCs using micro‐gravure R2R‐printed ZnO thin film as the ETL showed performance parameters comparable to those of spin‐coated ZnO thin‐film on the flexible substrate. Ma et al.^[^ ^54^ ^]^ demonstrated that by composition of the mixture solvent and the concentration of the AgNW solution, the surface tension, volatilization rate, and viscosity of the AgNW solution can be tuned to meet the requirements of the gravure printing process. Through careful solution formulation and printing processing optimization, transparent AgNW electrodes with a sheet resistance of 10.8 Ω sq^−1^ and a high light transparency of 95.4% can be achieved (**Figure** **5**a–c). As a result, high PCEs of 15.28% and 13.61% for small‐area (0.04 cm^2^) and large‐area (1.0 cm^2^) PM6:Y6‐based flexible OSCs were achieved, confirming good uniformity of the gravure‐printed AgNW electrode (PET/AgNWs‐GV). Although many researchers have successfully fabricated large‐area OSCs based on the gravure printing method, it is not an ideal method for the production of OSCs due to the following inherent characteristics: the printing roller needs to be completely replaced when coating different patterns, so this is a very expensive technique for volume production. In addition, R2R gravure printing of OSCs at high speed is difficult, as it requires additional time to spread out the printed solution.

### 2.4 Inkjet Printing

Inkjet printing (Figure 2d) is a non‐contact and high‐material utilization fabrication technology that facilitates the printing of various functional layers. The working principle usually comprises droplet ejection and formation, followed by positioning, spreading, and coalescence of droplets on the surface, and then solvent evaporation and solid film formation. The solution is dispersed by nozzles with fine control of the droplet size and trajectory, leading to a reproducible digital pattern with high resolution and no material loss. The solution of the inkjet printing method is usually required to have an appropriate viscosity and surface tension. Eggenhuisen et al.^[^ ^55^ ^]^ tried to add polystyrene to increase the viscosity of the solution to enhance the printability with a sacrifice of 20% of the device PCE. This is due to the poor morphology of inkjet droplets due to the increased viscoelasticity, although the addition of polystyrene with higher molecular weight or concentration results in higher solution viscosity. Ultimately, the P3HT:PCBM‐based OSCs device exhibited a PCE of 1.76% with active area of 0.805 cm^2^. Luo et al.^[^ ^56^ ^]^ developed an LBL inkjet printing at high temperature to obtain a homogenous and uniform film and achieved a PCE of 13.09% for the PM6:BTP‐BO‐4Cl‐based devices (Figure 5d,e). They demonstrated that both the molecular aggregation and vertical phase separation were highly printing temperature dependent. Increasing printing temperature can effectively suppress excess aggregation and improve the crystalline, but it also leads to a homogenous vertical phase separation from surface to bottom, which is adverse for charge collection. The merit of inkjet printing is that it can realize attractive the thin printed film with complex or varying patterns. However, the solution needs low viscosity (4–30 cP), high surface tension (typically 435 mN m^−1^), and the slow printing speed that inhibits the application of high throughput on printing large‐area OSCs.

## 3 Materials Requirements for a Large‐Area Active Layer

**Figure 6:**
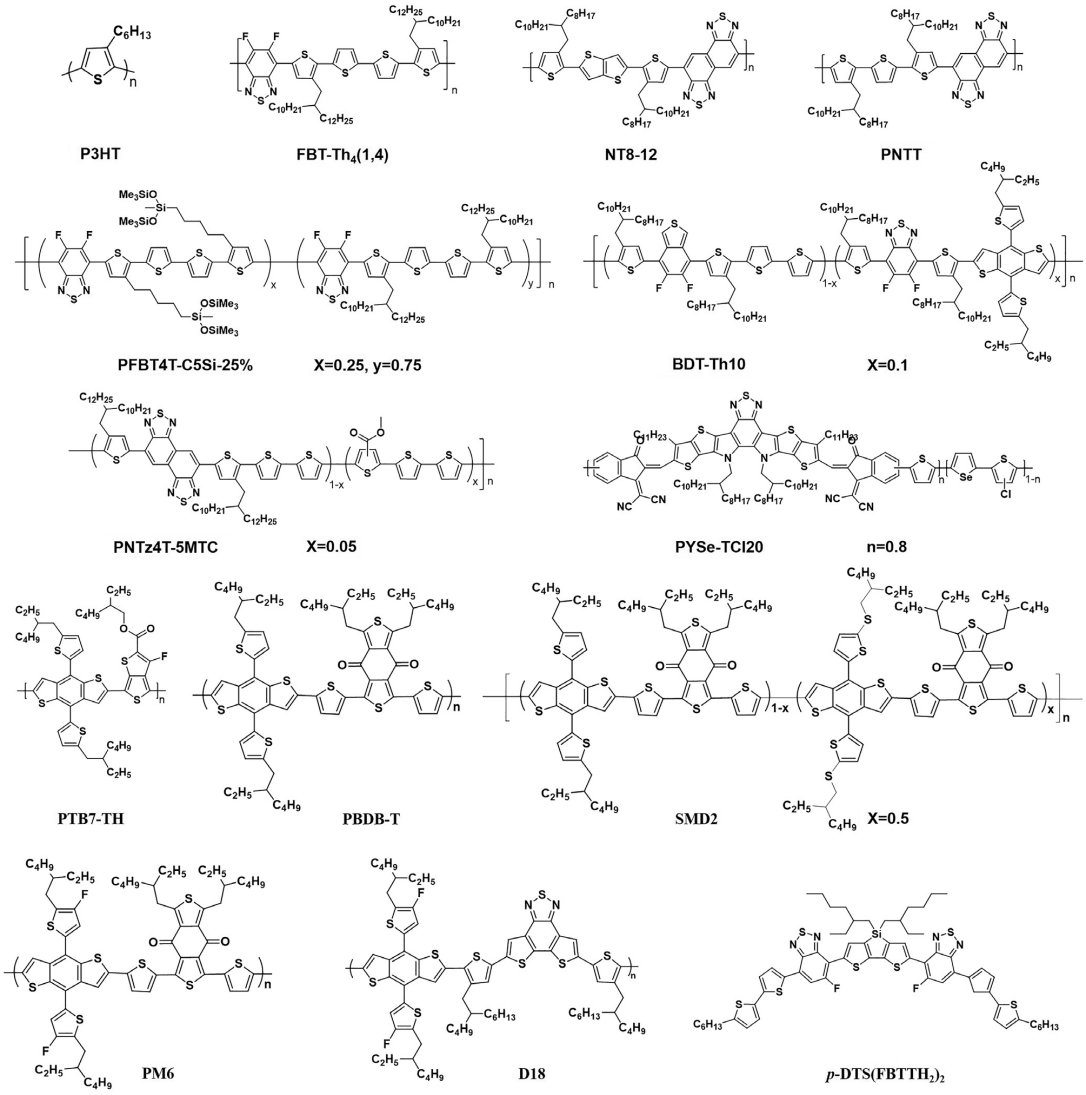
The chemical structures of high‐performance donor polymers that are suitable for thick large‐area active layers.

**Figure 7:**
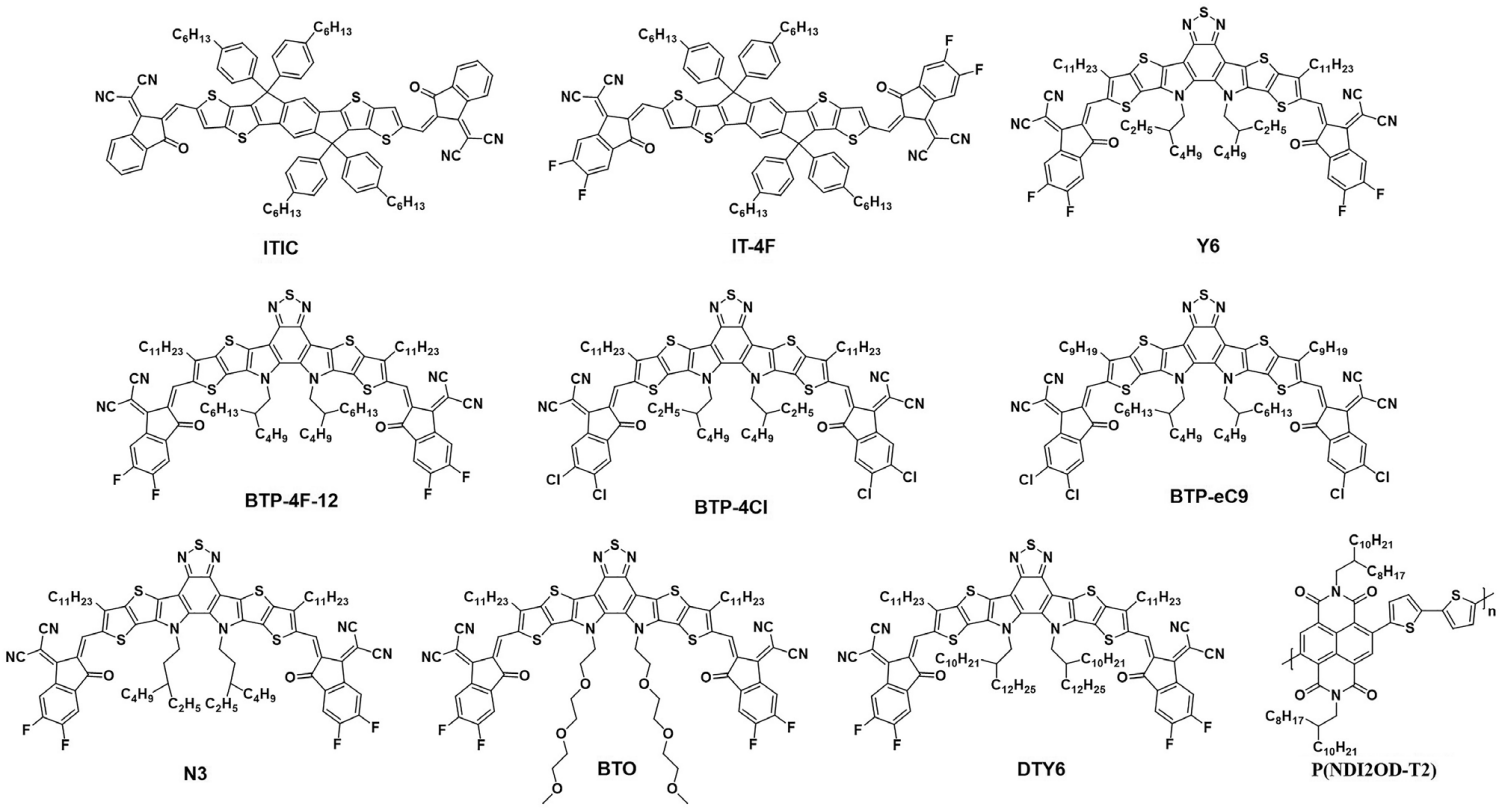
The chemical structures of high‐performance small‐molecule acceptors that are suitable for thick large‐area active layers.

The photoactive layer is the most critical component in large‐area OSCs. This active layer is normally prepared by the spin‐coating method with a thin‐film thickness of ≈100 nm due to the inherent low charge‐carrier mobility and strong exciton binding energy of organic materials.^[^ ^57^ ^]^ At present, the PCEs of large‐area OSCs are still far lower than that of the small‐area OSCs due to the following reasons: 1) there are a lot of point defects in large‐area organic films, which are prone to form large leakage currents, and small variation in the film thickness induces a relatively large difference in OSCs performance with low reproducibility; 2) the morphology of the large‐area active layer is difficult to control because of the different solvent evaporation and fluid mechanics behaviors of spin‐coating and scalable coating techniques; 3) in large‐area printing processes, NFAs exhibit different packing and crystallization behaviors, leading to insufficient charge transport. The active layer, as the most critical functional layer in OSCs, greatly affected these factors and the PCEs of large‐area devices. The requirement for the OSCs materials are as follows: 1) the active layer materials should have favorable thickness insensitivity to prepare high‐performance large‐area OSCs; 2) the organic materials should have regular molecular packing with face‐on orientation to provide efficient charge transfer; 3) the organic materials should have good solubility and crystallinity in eco‐compatible solvent to ensure green production in the future. In this section, we will provide a detailed introduction to these state‐of‐the‐art material systems that are adaptable to the large‐area preparation. The chemical structures mentioned in this section are shown in **Figures** **6** and **7** and the performances of corresponding OSCs devices are summarized in **Tables** **2** and **3**.

**Table 2:** The performance of OSCs devices with thick active layers

Active layer	Thickness [nm]	*V*_oc_ [V]	*J*_sc_ [mA cm^−2^]	FF [%]	PCE [%]	References
FBT‐Th_4_(1,4):PC_71_BM	230	0.76	16.2	62.1	7.64	[57]
	440	0.75	15.1	57.7	6.53	[57]
PFBT4T‐C5Si‐25%:PC_71_BM	270	0.76	17.92	74.61	10.39	[65]
	420	0.76	19.08	74.12	11.09	[65]
	660	0.75	18.32	71.47	10.15	[65]
NT812:PC_71_BM	340	0.72	19.09	72.85	10.33	[66]
	1000	0.72	18.12	62.15	8.35	[66]
PNTT:PC_71_BM	280	0.77	20.2	71.8	11.3	[67]
	1050	0.75	19.9	59.6	9.0	[67]
PM6:IT‐4 F	285	0.83	22.6	64.8	12.2	[71]
PM6:Y6	250	0.82	27.1	62.8	14.1	[72]
	300	0.82	26.5	62.3	13.6	[72]
PM6:BTP‐4Cl	328	0.84	27.5	64.9	15.0	[74]
	1020	0.834	27.3	53.1	12.1	[74]
PM6:BTP‐eC9	305	0.831	29.26	67.33	16.35	[76]

**Table 3:** The performance of OSCs devices with large‐area layers

Active layer	Area [cm^2^]	*V*_oc_ [V]	*J*_sc_ [mA cm^−2^]	FF [%]	PCE [%]	References
BDT‐Th10:PC_71_BM	58.5	7.527	1.55	66.34	7.74	[77]
PNTz4T‐5TMC:PC_71_BM	54.45	7.970	1.34	61	6.61	[78]
PBDB‐T:PYSe–TCl20:PTClo‐Y	1.21	0.908	22.3	68.1	13.81	[79]
PTB7‐Th:*p*‐DTS(FBTTH_2_)_2_:PC_71_BM	10	1.43	7.65	53.25	5.82	[80]
	20	2.71	3.89	49.19	5.18	[80]
PM6:Y6:BTO:PC_71_BM	36	10.02	2.01	70.81	14.26	[81]
PM6:N3:P(NDI2OD‐T2)	58.5	8.61	2.43	67.1	14.04	[82]
T1:BTP‐4 F‐12	1.07	0.85	24.2	70	14.4	[83]
PM6:DTY6	18	5.11	3.89	72.5	14.4	[84]
PBF–QxF:Y6	0.09	0.76	20.39	62	9.63	[86]

### 3.1 Materials Applicable to a Thick Active Layer

High‐performance OSCs with thick active layers are essential for large‐scale production, because they can almost fully utilize the sunlight and is more compatible with R2R processing. However, the PCEs of thick‐film OSCs are generally inferior to their thin‐film devices, which is due to the serious charge recombination caused by the low mobility and poor morphology in the thick active layer.^[^ 58, 59 ^]^ An effective strategy to develop high‐performance thick‐film OSCs is to design and synthesize novel active materials with high charge mobility. The high mobility of the donors facilitates hole transport, thus endowing the active layer with high thickness‐insensitive properties. Due to the high crystallinity and hole mobility (*μ*_h_, 5 × 10^−3^ cm^2 ^V^−1^s^−1^) of P3HT, the P3HT‐based system shows good film thickness insensitivity.^[^ ^60^ ^]^ However, its high‐lying highest occupied molecular orbital (HOMO) level and narrow absorption edge limit the further improvement of device performance.^[^ ^61^ ^]^ Therefore, numerous research has focused on the development of donor materials with high crystallinity and planarity to broaden the absorption range and downshift the HOMO energy level by side‐chain engineering. Fluorine‐substituted benzothiadiazole (FBT) is a strong electron‐deficient unit for constructing low bandgap polymer donors with deep HOMO levels.^[^ 62, 63, 64 ^]^ The existent fluorine atoms on the FBT unit can enhance the intramolecular and intermolecular interactions, resulting in the corresponding polymer donors with better backbone planarity and crystallinity. Chen et al.^[^ ^57^ ^]^ reported a D–A copolymer FBT‐Th_4_(1,4) using 5,6‐difluorobenzothiazole unit as the A‐unit and quarter‐thiophene with solubilizing alkyl chains attached on the two terminal thiophene rings as the D‐unit. FBT‐Th_4_(1,4) has a strong temperature‐dependent aggregation and forms pre‐aggregation in solution, making the hole mobility up to 1.92 cm^2 ^V^−1^s^−1^. As a result, the FBT‐Th_4_(1,4):PC_71_BM‐based device presented the highest PCE of 7.64% at a film thickness of 230 nm and 6.53% at 440 nm in the spin‐coated OSCs, showing a high thickness‐independent characteristic. Moreover, Chen et al.^[^ ^65^ ^]^ developed a side‐chain random copolymer, PFBT4T‐C5Si‐25%, with 25% content of siloxane‐terminated side chain in conjunction with the 2‐decyltetradecyl side chain. The introduction of siloxane‐terminated side chain enhanced the face‐on orientation and resulted in favorable 3D hole transport in thick films, such that the fill factor becomes stabilized at 74% as the short‐circuit current density (*J*_SC_) slightly increases with greater film thickness. The PFBT4T‐C5Si‐25%:PC_71_ BM‐based spin‐coated OSCs with 270, 420, and 600 nm‐thick active layer exhibit the PCEs of 10.39%, 11.09%, and 10.15%, respectively. Naphtho[1,2‐c:5,6‐c]bis[1,2,5]thiadiazole (NT), a derivative of benzo[2,1,3]‐thiadiazole (BT), contains two fused BT units whose enlarged planeness enhance the charge mobility and interchain packing, which shows great potential in the preparation of highly efficient thick‐film OSCs. Huang et al.^[^ ^66^ ^]^ developed a novel polymer donor, NT812, with NT as the A‐unit and 2,5‐bis(3‐alkylthiophen‐2‐yl)thieno[3,2‐b]thiophene (BTTT) as the D‐unit. The centrosymmetric BTTT and NT units with large planarity and *π*‐conjugation length can enhance the crystallinity and charge mobility of NT812, and the hole mobility of BHJ film based on NT812 is as high as 2.7 × 10^−2^ cm^2^ V^−1^ s^−1^. At an optimal thickness of 340 nm, the NT812:PC_71_BM‐based OSCs show a remarkable efficiency of 10.33% in spin‐coated OSCs, and the PCE remains at 8.35% with an active layer thickness of 1000 nm. It is exciting that when using environment‐friendly solvent (*o*‐xylene) processing, the device efficiency exceeds 10% at the film thickness of 300 nm. Afterward, Huang et al.^[^ ^67^ ^]^ developed a novel polymer donor, PNTT, with NT as the A unit and thiophene flanked with alkylthiophene as the D unit. The OSCs based on PNTT:PC_71_BM exhibited the best PCE of 11.3% with an active layer thickness of 280 nm and PCEs greater than 10% with active layer thicknesses of 150–660 nm. The PCE remained higher than 9% at a film thickness of 1050 nm, which is one of the highest PCE values for thick‐film OSCs with BHJ film thicker than 1 μm. The excellent device performance at different film thicknesses was attributed to deep HOMO energy levels and high hole mobility of PNTT (2.7 × 10^−2^ cm^2 ^ V^−1^ s^−1^).

Even though the NFAs exhibit favorable photoelectric properties and high efficiency in the OSCs, they are quite sensitive in the thick film due to the lower electronic mobility.^[^ 68, 69 ^]^ Thus, to overcome this obstacle, many researchers have designed a series of NFAs with strong aggregation and high mobility. Hou et al.^[^ ^70^ ^]^ developed a novel acceptor IT‐4F by fluorinating the well‐known acceptor ITIC. The fluorination effectively decreased the energy bandgap of the acceptor, resulting in a broader range absorption as well as deeper HOMO and lowest unoccupied molecular orbital energy levels. Furthermore, the fluorination of the acceptor induced the formation of more ordered intermolecular arrangements and enhanced charge‐carrier mobility in the BHJ films. As a result, the PM6:IT‐4F‐based spin‐coated thin‐film OSCs achieve a PCE of 13.5% by optimizing the active layer thickness and the PCE remains at 12.2% with the active layer thickness of up to 285 nm.^[^ ^71^ ^]^ Zou et al.^[^ ^72^ ^]^ developed an NIR absorption NFA of Y6, that employs a ladder‐type multi‐fused ring central unit with an electron‐deficient BT core to finely tune its absorption and electron affinity. The resulting PM6:Y6‐based thin‐film spin‐coated OSCs exhibited a record efficiency of 15.7%. In addition, those devices also maintained a PCE of 14.1% and 13.6% with a thicker active layer of 250 and 300 nm, respectively. Since then, Y6 and its derivatives have received wide attention due to their outstanding photovoltaic performance, and are expected to become the star acceptor materials for OSCs. Hou et al.^[^ ^73^ ^]^ further developed a chlorinated low bandgap acceptor BTP‐4Cl by replacing the halogen atoms of Y6, which exhibits an extended optical absorption and meanwhile displays a higher voltage than its fluorinated counterpart in the devices. As a result, the PM6:BTP‐4Cl‐based spin‐coated 328 nm‐thick OSCs achieved a remarkable PCE of 15.0% and maintained a PCE of 12.1% with an active layer thickness of 1020 nm.^[^ ^74^ ^]^ Subsequently, Hou et al.^[^ ^75^ ^]^ continued to optimize the edge of alkyl chain of BTP‐4Cl, and synthesized (2,2′‐((2Z,2′Z)‐((12,13‐bis(2‐butyloctyl)‐3,9‐dinonyl12,13‐dihydro‐[1,2,5]thiadiazolo[3,4‐e]thieno[2″,3″:4′,5′]thieno[2′,3′:4,5]pyrrolo[3,2‐g]thieno[2′,3′:4,5]thieno[3,2‐b]indole‐2,10‐diyl)bis (methaneylylidene))bis(5,6‐dichloro‐3‐oxo‐2,3‐dihydro‐1H‐indene‐2,1‐diylidene))dimalononitrile) (BTP‐eC9) by shortening the *n*‐undecyl (C11) to *n*‐nonyl (C9). In comparison with BTP‐eC11, the BTP‐eC9 with shorter alkyl chains shows suitable solubility, decreased Urbach energy, and enhanced electron mobility. The PM6:BTP‐eC9‐based spin‐coated OSCs with a 110 nm active layer show PCE of 18.32% and the device with a 305 nm‐thick film delivers a PCE of 16.35%, which is among the highest values reported.^[^ ^76^ ^]^

### 3.2 Materials Applicable to Large‐Area Printing

For small‐area devices, the dying of wet active layer film is due to the centrifugal effect during spin‐coating. However, the film formation and drying behaviors in scalable coating methods are different. Therefore, it is of great importance to seek suitable active layer materials to optimize morphology for large‐area device fabrication. Synthesis of random terpolymer donors was demonstrated to be a good strategy for realizing high‐performance large‐area active layers. Son et al.^[^ ^77^ ^]^ developed a high‐performance random terpolymer BDT‐Th10, by introducing 10% feed molar ratio of a benzodithiophene moiety into a highly crystalline copolymer BDT‐Th0. The BDT‐Th0 polymer formed irregular large aggregates in the BHJ films, whereas BDT‐Th10 showed an appropriate packing structure and phase‐separation morphology without substantial agglomeration. Therefore, the BDT‐Th10:PC_71_BM‐based device achieved a high PCE of 7.74% in a large‐area module (58.5 cm^2^) with 350 nm film thickness, indicating that the BDT‐Th10 random terpolymer is potentially applicable to the large‐area OSCs. Shin et al.^[^ ^78^ ^]^ developed random terpolymers of PNTz4T polymer with different ratios of bithiophene substituted with methyl thiophene‐3‐carboxylate (MTC). With the incorporation of MTC moiety, the random ternary polymer has better solubility, lower crystallinity, smaller aggregation tendency, and weaker intermolecular interaction. As a result, the optimal random terpolymer PNTz4T‐5MTC‐based OSCs exhibited the PCE of 6.61% in a 54.45 cm^2^ OSC module using the non‐halogenated solvent. Chen et al.^[^ ^79^ ^]^ developed random terpolymer acceptors PYSe–TClx with 3‐chlorothiophene (TCl) embedded into PYSe randomly (where *x* stands for the molar ratio of TCl unit). As random copolymerization enables significantly reduced aggregation and up‐shifted energy levels of the terpolymer PYSe–TClx system, the all‐polymer OSCs based on PYSe–TCl20 exhibit an outstanding PCE of 15.26%. More importantly, precisely regulating the aggregation of blend film by the synergistic effect of terpolymerization with the non‐conjugated backbone approach can effectively optimize the film quality during blade‐coating process. Consequently, the optimized system with a large‐area of 1.21 cm^2^ achieved a high PCE of 13.81% and 13.21% using chloroform (CF) and *o*‐xylene solvents, respectively. In addition, the ternary strategy also proved to be an effective way to improve the PCE of large‐area OSCs. Wei et al.^[^ ^80^ ^]^ introduced highly crystalline small‐molecular donor *p*‐DTS(FBTTh_2_)_2_ into the blend of PTB7‐Th donor and PC_71_BM acceptor, effectively improving the molecular crystallinity and face‐on orientation in the BHJ film without sacrificing the solution processability. Consequently, the ternary blend with 15% weight ratio *p*‐DTS(FBTTh_2_)_2_‐based large‐area flexible modules achieved the PCEs of 5.82% and 5.18%, with the active area 10 and 20 cm^2^, respectively. Li et al.^[^ ^81^ ^]^ developed a unique guest‐assisted assembly strategy by introducing guest molecular BTO containing an amphiphilic oligo(ethylene glycol) chain, which showed good solubility in a high‐boiling‐point green solvent and excellent compatibility with the host‐component Y6, assisting Y6 to crystallize in the green solvent. Benefiting from this efficient manipulation of the host molecular structures, the PM6:Y6:20%BTO:PC_71_BM‐based 36 cm^2^ large‐area OSC module demonstrated a record PCE of 14.26% with processed from non‐halogenated paraxylene. Importantly, the corresponding OSCs retain 75% of their initial efficiency following 1000 h of operation at the maximum power point under 100 mW cm^−2^ white light‐emitting diode illumination. Son et al.^[^ ^82^ ^]^ developed high‐performance large‐area OSC module by introducing the second acceptor P(NDI2OD‐T2) into PM6:N3 active layer. A small amount of the second acceptor forms intricate P(NDI2OD‐T2) channels in the donor domain with cascade‐energy‐level alignment, which practically assist exciton dissociation in the donor domain and improve the electron‐transporting pathway. As a result, the PM6:N3:P(NDI2OD‐T2)‐based OSC module with an active area of 58.5 cm^2^ showed a higher PCE of 14.04% than that of the PBDB‐T‐2F:N3 device (12.59%), which is among the highest efficiency reported in large‐area OSCs with active area above 50 cm^2^. In addition, designing high‐performance acceptor materials is another way to improve the PCEs of large‐area OSCs. Hou et al.^[^ ^83^ ^]^ synthesized high‐efficiency NFA BTP‐4F‐12, which has longer alkyl side chains (2‐butyloctyl) than BTP‐4F‐8 (2‐ethylhexyl). Owing to the enhanced lamellar stacking of BTP‐4F‐12 with modified alkyl chains, better charge transport was obtained in the corresponding devices. More importantly, the BTP‐4F‐12 showed improved solubility in some low‐toxic solvents, and the tetrahydrofuran (THF)‐processed devices via the blade‐coating technique yielded a high PCE of 14.4% with an active area of 1.07 cm^2^. Huang et al.^[^ ^84^ ^]^ designed an NFA DTY6 with long‐branched alkyl chains (2‐decyltetradecyl, 2‐DT) on TPBT (dithienothiophen[3.2‐b]‐pyrrolobenzothiadiazole) central unit. The long‐branched alkyl chain was employed to improve the solubility and ensure the solvent‐processing ability, and the steric hindrance effect of long‐branched alkyl chain was used to suppress the molecule‐excessive aggregation. Encouragingly, the 18 cm^2^ large‐area OSC module based on the non‐halogenated solvent *o*‐xylene‐processed PM6:DTY6 active layer exhibited an outstanding PCE of 14.4%.

For employing the R2R printing technique for the fabrication of large‐area OSCs, it is important to use novel R2R‐compatible materials or mold the properties of the existing active layer materials formulations according to the technical requirements. In this regard, designing materials that have high mobility and are thickness insensitive is significantly important but has not yet been solved. Andreasen et al.^[^ ^85^ ^]^ explored in situ small and wide‐angle X‐ray scattering study of two polymers that are relevant for organic photovoltaics and revealed that fast‐drying polymers which are influenced less by drying temperature or substrate inhomogeneities are better suited for R2R coating. Chang et al.^[^ ^86^ ^]^ developed two D–A‐type quinoxaline‐based donor polymers with multiple fluorine atoms, PB–QxF and PBF–QxF. Owing to the positive contributions of the two additional fluorine atoms on the BDT unit, the PBF–QxF exhibited enhanced charge mobilities with a lower HOMO energy level, resulting in a higher PCE than that of PB–QxF. In addition, PBF–QxF‐based devices showed a remarkable thickness tolerance to PCE of up to 350 nm, exhibiting the highest PCE of 9.63% in R2R‐processed additive‐free OSCs.

In general, realizing efficient large‐area OSCs necessitates the development of a BHJ film with nanoscale phase separation in the thick active film and active layer materials that are not easily aggregated during the large‐area printing processes, which enable high and balanced charge‐carrier transport. A number of strategies have been developed, including the design of high‐mobility active layer materials, random copolymerization of donor polymers, and the introduction of a third component to control the nanoscale morphology and achieve high solution processability. These strategies have created room for improving the efficiency of large‐area OSCs.

## 4 Materials Requirements for a Large‐Area Interface Layer

Printable interface layers that dominate charge transport and collection in the OSCs also are crucial for obtaining superior high‐throughput printed large‐area OSCs. The printed interface layers are required to have the following characteristics of excellent electron‐transport performance, good film‐forming properties, and good Ohmic contacts. At present, several interface layers, especially CIL materials cannot be prepared by printable techniques due to their low electron mobility.^[^ ^87^ ^]^ Furthermore, to fabricate high‐quality large‐area interface layers, the fluidic characteristics in the printing process of CILs must be carefully regulated. Therefore, it is crucial to develop new materials and optimize the solution processing of CILs. In this section, we will briefly introduce the progress of new interface layer materials and the methods of regulating fluid properties.

At present, the lack of printable CIL has greatly impeded the pace toward practical production of OSCs, and thus the development of a new CIL with high electron mobility is in urgent demand. Hou et al.^[^ ^88^ ^]^ reported an organic molecule based on naphthalene diimide (NDI), namely (*N*,*N*‐dimethylamino)propyl naphthalene diimide (NDI‐N), as a printable CIL for OSCs. Compared with NDI‐Br (*N*,*N*‐dimethyl‐*N*‐ethylammonium)propyl naphthalene diimide, NDI‐N can efficiently extract electrons from both the NFA and the polymer donor. In addition, NDI‐N combines the merits of high crystallinity and good film‐forming properties, endowing the semiconductor with excellent electron‐transport properties and good processability. More importantly, when the NDI‐N thickness changed from 5 to 30 nm, the PCE remained at over 11.6%, indicating the outstanding tolerance to thickness variation of NDI‐N. Ultimately, a large‐area OSCs of 1.0 cm^2^ was fabricated with the blade‐coated NDI‐N and an impressive PCE of 13.2% was achieved. Xu et al.^[^ ^89^ ^]^ demonstrate a new strategy of tailoring the end‐capping unit ITIC to develop the organic small molecule S‐3 as CIL for achieving high‐performance OSCs. The excellent electron‐accepting capacity, suitable energy level, and good film‐forming ability of S‐3 molecule endowed the blade‐coated S‐3‐based OSCs with a PCE of 16.02%, which is comparable to that of the spin‐coated device (16.6%). Furthermore, the large electrostatic potential difference between the S‐3 and the polymer donor could induce the intermolecular electric field, which promotes the exciton dissociation for an additional charge generation.

In addition to developing novel interface materials, their fluid mechanics also need to be carefully studied. Hou et al.^[^ ^90^ ^]^ developed a new method for simultaneously manipulating fluidics of the sol–gel ZnO precursor and optimizing the processability of the ZnO layer for flexible OSCs. They found that by adopting the Lewis bases with varied ionization equilibrium constants (pK_b_) in the sol–gel method, the reaction process in the solution state, the annealing temperature, the fluidics, and the proportion of the polar facets can be effectively modulated. The moderate pK_b_ of *n*‐propylamine (PA, pK_b_ = 3.43) compared to ethanolamine (EA, pK_b_ = 4.50) and triethylamine (TEA, pK_b_ = 3.28) produces a moderate reaction rate in the sol–gel process (EA < PA < TEA). The gentle but complete reaction makes the PA‐based solution exhibit suitable fluidic dynamics and the low necessity for annealing temperature (130 °C), which prevent the formation of bumps or coffee rings, and enable the preparation on polyethylene naphthalate/ITO. Thus, the 1.0 cm^2^ flexible OSCs with the PA–ZnO exhibits good photostability and an outstanding PCE of 16.71%.

## 5 Morphology Optimization Strategies

The photovoltaic performance of the OSCs is closely related to the morphology of the active layer, as the molecular packing and domain size have critical effects on the exciton dissociation and charge transport. The morphology of the blend film is determined by the inherent material properties and the film‐formation processing. Due to the different film‐formation mechanisms between the spin‐coating and scalable coating, the traditional morphology optimization strategy in spin‐coating may not be adaptable for large‐area OSCs. Thus, it is of great importance to seek the morphology optimization strategy for large‐area fabrication. Several methods have been proposed to regulate the morphology, including solvent and additive engineering, coating speed/temperature regulating, exploiting LBL‐processing method, and fluid mechanical research.

### 5.1 Solvent Engineering

The successful selection of the active layer solvent is the prerequisite for high‐efficiency OSCs. Using suitable solvents can improve the morphology with a smooth surface and high crystallinity. Hou et al.^[^ ^91^ ^]^ obtained a device efficiency of 11.7% by blade‐coating with THF/isopropyl alcohol solvent, which is comparable to the performance of spin‐coating device (12.0%), while the efficiency of *o*‐xylene/1‐phenylnaphthalene (PN)‐processed solvent decreased significantly. They found that when using the blade‐coating method, due to the high‐boiling‐point *o*‐xylene/PN (144/325 °C), the drying process will be greatly prolonged, and the phase‐separation morphology of the film changed greatly, resulting in the formation of aggregates of over several hundred nanometers on the film (the surface roughness increases to 20 nm), and serious non‐radiative recombination. In addition, the THF‐processed PBTA‐TF:IT‐M large‐area device (1.0 cm^2^) still maintained a PCE of 10.6%. Min et al.^[^ ^92^ ^]^ developed an effective co‐solvent (CF/CB) strategy to finely tune the film formation for achieving suitable BHJ morphology. They illustrate that the film‐formation process is precisely controlled when using the co‐solvent, and the blend showed high domain purity with suitable phase‐separated networks. As a result, a PCE of 16.17% was achieved in the blade‐coated PM6:Y6‐2Cl device without any post‐treatments, which is much higher than those of CF‐ or CB‐processed devices (14.08% and 11.44%, respectively).

In addition, halogenated solvents such as *o*‐dichlorobenzene (*o*‐DCB), CB, CF have been widely used and demonstrated to be the most efficient solvents for fabricating high‐performance OSCs. Unfortunately, these solvents are harmful to the natural environment and human beings, which are prohibited according to industry standards. Therefore, the development of green solvent‐processing technology is greatly needed for the commercialization of large‐area OSCs. **Table** **4** summarizes the physical properties (toxicity, solubility, volatility and boiling point, etc.) of commonly used green solvents compared to halogenated solvents. It is well known that photoactive layer materials are usually organic conjugated materials with a highly rigid structure, so they have poor solubility in green solvents. Therefore, molecular design strategies that finely turn the structure of conjugated materials for increasing their solubility in the green solvent are needed. Lim et al.^[^ ^93^ ^]^ developed a novel NFA T2‐OEHRH with an asymmetric structure. The introduction of asymmetric alkyl side chains onto rhodanine end groups can effectively suppress excessive self‐aggregation/crystallization and substantially improve solubility in green solvents without sacrificing optoelectrical properties. Therefore, the OSCs based on PTB7‐Th:EH‐IDTBR:T2‐OEHRH treated with toluene showed a uniform and favorable morphology and gave a high PCE of 9.32% in large‐area modules (55.5 cm^2^). Chang et al.^[^ ^94^ ^]^ synthesized new TPD‐T2 (2,5‐dithienyl‐thieno[3,4‐c] pyrrole‐4,6‐dione) acceptor moiety based donor polymers TPD‐*n* (*n* = 1–3). All polymers readily dissolve in green solvent *o*‐xylene, and the corresponding blend films can be processed in ambient to fabricate OSCs with PCEs of 12%–14%. More importantly, the TPD‐3F:IT‐4F‐based large‐area OSC module (20.4 cm^2^) processed by blade‐coating using green solvent *o*‐xylene under ambient conditions showed an outstanding PCE of 10.4%. Regulating suitable single or mixed green solvents to process the original material can also achieve the desired effect for fabricating eco‐friendly OSCs. Huang et al.^[^ ^95^ ^]^ replaced the CB/DIO solvent with the single‐component green solvent 2‐methylanisole (2‐MA) to process the PTB7‐Th:PC_71_BM active layer. The solvent 2‐MA is nontoxic and selectively dissolves PTB7‐Th and PC_71_BM (solubility of ≈15 mg mL^−1^ for PTB7‐Th and >25 mg mL^−1^ for PC_71_BM). The selective solubility of 2‐MA in terms of PTB7‐Th and PC_71_BM is favorable for the ideal morphology formation as in the case of CB/DIO. As a result, a 16 cm^2^ large‐area OSC device processed by blade‐coating with a PCE of 7.5% was also obtained with 2‐MA, indicating that 2‐MA has good processability.

**Table 4:** The physical properties of common organic solvents

Organic solvents	Toxicity (expressed by the size of median lethal dosea))	Solubility	Volatility	Boiling Point [°C]
CF	908	High	High	61.7
CB	1100	High	Low	132.2
*o*‐DCB	500	High	Low	180.5
1,2,4‐Trichlorobenzene	756	High	Low	214.1
Toluene	5000	Medium	Medium	110.6
o‐xylene	4300	Medium	Low	144.8
1,2,4‐Trimethylbenzene	3800	Medium	Low	168.9
Anisole	3700	Low	Low	153.8
Diphenyl ether	2450	Low	Low	257.9
2‐MA	N/A	Low	Low	171.1
Tetrahydrofuran	1650	Low	High	66.0
2‐Methyltetrahydrofuran	5720	Low	High	79.9
Cyclopentyl methyl ether	1000‐2000	Low	Medium	105.3
Carbon disulfide	2780	Low	High	46.2
N‐Methyl‐pyrrolidone	3914	Low	Low	202.0
Acetone	5800	Low	High	56.5
Ethyl acetate	5620	Low	High	73.9
Isopropyl alcohol	5000	Low	High	82.5
Water	90 000	Low	Medium	100.0

a) Median lethal dose values (in mg Kg^−1^) for various organic solvents. The values were collected from relevant literature.^[^ 130, 131 ^]^

### 5.2 Additive Engineering

**Figure 8:**
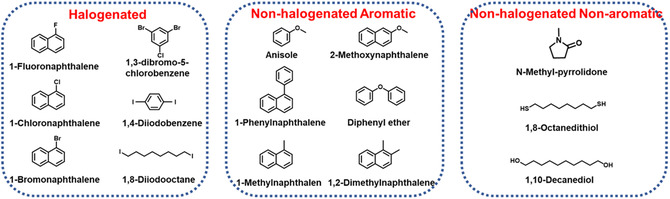
The chemical structures of common additives.

Additive engineering can also effectively optimize the blend film morphology by regulating the aggregation state, solubility, and drying kinetics. The chemical structures of common additives are shown in **Figure** **8** and the performance of corresponding OSCs devices are summarized in **Table** **5**. Xie et al.^[^ ^96^ ^]^ added 1,2‐dimethylnaphthalene (DMN) additive to PM6:Y6 solution for improving the film crystallinity. With the addition of 0.5% volume fraction of the DMN, the Y6 absorption peaks are blue‐shifted significantly, indicating that the aggregation of Y6 was inhibited. In addition, high‐boiling‐point non‐halogenated solvent additive DMN can induce more nucleation sites and enhance the crystallinity of both PM6 and Y6. Consequently, the OSCs with an effective area of 1.0 cm^2^ showed a high PCE of 13.87%. Apart from this, the polymer molecule additives can also regulate molecular crystallization. Huang et al.^[^ ^97^ ^]^ have successfully added a small amount of n‐type polymer (N2200) in the PM6:Y6 active layer to regulate film morphology. Benefiting from the better miscibility between Y6 and N2200, the strong aggregation of NFA during the blade‐coating process is efficiently suppressed, leading to blend films with reasonably small domains, which in turn ensure efficient charge extraction and suppressed charge recombination. Finally, the large‐area OSC device (1.0 cm^2^) based on PM6:Y6:10%N2200 active layer exhibited a PCE of 15.1%. Yuan et al.^[^ ^98^ ^]^ investigated the influence of three additives, DIO, 1,8‐octanedithiol (ODT) and 1‐chloronaphthalene (CN), on the morphology of blade‐coated PBDB‐T:ITIC devices. Although the DIO‐based device obtained a high PCE of 9.87%, the corresponding device stability was poor, due to the dissociation of residual DIO into iodooctane and iodine radicals under illumination. Similarly, the efficiency gain of CN‐based devices was also limited. Impressively, owing to the enhanced crystallization and small and pure domains, devices with ODT additive not only possessed higher PCE (10.20%), but also exhibited better device stability. In addition, the ODT‐based large‐area device (0.56 cm^2^) prepared by blade‐coating also exhibits a high PCE of 8.59%. Meng et al.^[^ ^99^ ^]^ prepared PTB7‐Th:PC_71_BM‐based large‐area OSC module by incorporating *N*‐methyl‐2‐pyrrolidone (NMP) into the toluene green solvent. They added 3% volume fraction of NMP to improve good solubility for conjugated polymers/fullerenes and obtain favorable BHJ morphology. Through the combination of morphological manipulation of active layer and interfacial engineering, the highest achievable PCE of the blade‐coated 216 cm^2^ large‐area OSC module reaches 5.27%.

**Table 5:** The performance of large‐area OSCs devices with different additives

Active layer	Additive	Area [cm^2^]	*V*_oc_ [V]	*J*_sc_ [mA cm^−2^]	FF [%]	PCE [%]	References
PM6:Y6	DMN	1.0	0.836	24.48	67.87	13.87	[96]
PM6:Y6	N2200	1.0	0.83	26.2	69	15.1	[97]
PBDB‐T:ITIC	ODT	0.56	0.87	16.80	58.98	8.59	[98]
PTB7‐Th:PC_71_BM	NMP	216	0.85	24.07	64.00	13.09	[99]

### 5.3 Coating Speed/Temperature

**Figure 9:**
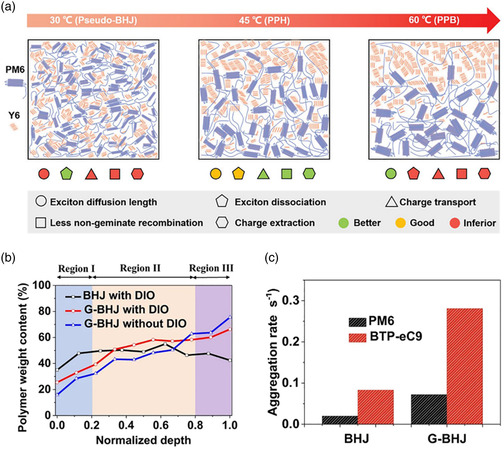
a) The evolution of morphological characteristics and fundamental processes as a function of baseplate temperature. Reproduced with permission.^[^ ^101^ ^]^ Copyright 2021, Wiley‐VCH. b) Variation of polymer weight content of BHJ with DIO, graded BHJ (G‐BHJ) without DIO and G‐BHJ with DIO films throughout the whole film. c) Aggregation rate of PM6 and BTP‐eC9 in optimized BHJ and G‐BHJ active layers. b,c) Reproduced with permission.^[^ ^104^ ^]^ Copyright 2021, Springer Nature.

The coating speed in the scalable coating method is generally considered to be the most crucial parameter, with a tremendous impact on the film thickness and quality. A faster coating speed will reduce the solution viscosity and drag a smaller amount of solution, causing solution convection and leading to poor film morphology. However, the slower coating speed makes the formation of low‐crystallinity films. Therefore, an appropriate coating speed will directly affect the film quality and device performance. Under the constant coating speed in the blade‐coating method, the film thickness gradually decreases along the coating direction as the consumption of solution, leading to substantial nonuniformity in the thickness. Meng et al.^[^ ^100^ ^]^ proposed the accelerated coating speed significantly improved the thickness uniformity of blade‐coated layers of polymer solar cells on an A4 glass substrate. As a result, when the acceleration is 10 mm s^−2^, the POD2T‐DTBT:PC_71_BM‐based device obtained a PCE of 3.64% with an active area of 108 cm^2^. In addition, the coating temperature also plays an important role in regulating the crystallization process. Min et al.^[^ ^101^ ^]^ systematically studied the influence of substrate temperature on the photovoltaic performance of PM6:Y6‐based devices using sequential blade‐coating deposition technology. They found that as the temperature increases, the morphology of the active layer has undergone a distinct evolution from the pseudo‐BHJ (30 °C) to a pseudo‐planar heterojunction (45 °C) and then to a pseudo‐planar bilayer (60 °C), leading to a non‐monotonic correlation between baseplate temperature and device performance (**Figure** **9**a). Due to the excellent vertical phase distribution of pseudo‐planar heterojunction, the blend film fabricated at 45 °C exhibits optimal vertical composition gradient and thus yielded a higher device performance than those of the active layers prepared at lower or higher baseplate temperatures.

### 5.4 LBL‐Processing Method

In the past 20 years, BHJ has been a common choice for researchers to process active layers. However, the formation of BHJ active layer is a complex process, which is strongly dependent on the conditions of donor and acceptor blend solution, resulting in undesirable vertical phase separation, and unfavorable charge transfer and collection. Moreover, during the coating process, the slow drying of donor and acceptor mixture wet film makes very different crystallization and intermixing dynamics, leading to completely different D–A phase‐separation morphologies. Compared with BHJ structure, the LBL deposition process has greater advantages in morphology regulation, such as the independent crystallization process of the donor and acceptor materials, and the more ideal vertical phase‐separation structures.^[^ 21, 102, 103 ^]^ Li et al.^[^ ^104^ ^]^ demonstrated a simple yet effective G‐BHJ‐formation strategy to optimize the morphology of the PM6:BTP‐eC9 active layer. They found that the sequentially deposited films display a clear graded polymer distribution from the top surface to the bottom (BTP‐eC9‐enriched at the top, and PM6‐enriched at the bottom), which is clearly distinguished from the BHJ film's vertical polymer distribution (Figure 9b). Furthermore, they observed that the aggregation rate of the acceptor in G‐BHJ film is higher than that in BHJ films (0.281 s^−1^ for G‐BHJ and 0.083 s^−1^ for BHJ), indicating that acceptor could rapidly aggregate in G‐BHJ film (Figure 9c). Ultimately, the non‐halogenated solvent (*o*‐xylene) enabled G‐BHJ OSCs‐based open‐air blade‐coated method to achieve a record PCE of 16.77%. Min et al.^[^ ^105^ ^]^ also proposed that LBL deposition process combined with a blade‐coating approach can facilitate high‐speed (maximum speed: 30 m min^−1^) fabrication of photoactive layers. The traditional BHJ method will lead to serious phase separation and huge roughness of the active layer under high‐speed coating. By balancing film‐formation kinetics with respect to the factors of baseplate temperature, solution concentration, and coating speed, they achieved highly efficient 1.0 cm^2^ solar cells (16.8%) and 7.5 cm^2^ solar module‐based (14.6%) PM6:T8 films fabricated at a coating speed of 30.0 m min^−1^. More importantly, the LBL‐processing strategy also leads to a decrease in the minimum sustainable price values, thus providing the potential for high‐throughput fabrication of thin‐film OSCs.

### 5.5 Fluid Mechanics

**Figure 10:**
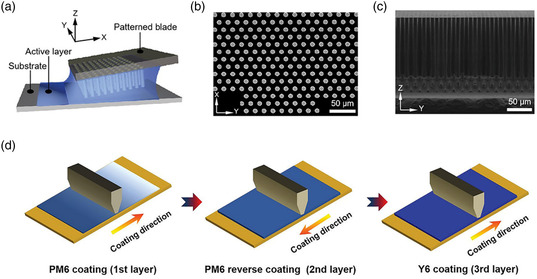
a) Schematic illustration of patterned blade coating. b) Typical SEM image of the circular patterned coating blade. c) Cross‐sectional SEM image of the circular patterned coating blade. a–c) Reproduced with permission.^[^ ^110^ ^]^ Copyright 2021, Wiley‐VCH. d) Schematic diagram of RS‐LBL active layer fabrication. Reproduced with permission.^[^ ^111^ ^]^ Copyright 2022, Wiley‐VCH.

The fluid flow during the coating process is also crucial due to the crystalline and anisotropic nature of non‐fullerene photoactive materials. The film morphology depends heavily on the fluid mechanical in the scalable coating process as the conformation and nonequilibrium assembly of the conjugated organic molecules could be affected by both the shearing flow and the extensional flow. The shearing flow promotes the crystallization kinetics and enhances the formation of ordered structures of the polymer. The extensional flow could effectively induce crystallization via stretching polymer chains. In addition, the shear speed also affects the fluid flow. At a low coating speed, evaporation drives the capillary flow, and the solute is transported to the three‐phase contact line, which is called the evaporation regime.^[^ ^106^ ^]^ During this regime, the solvent evaporation speed is faster than the coating speed, showing a thicker film as the slower coating speed. Through increasing the coating speed, the fluid flow is similar to laminar flow, with different shear strain distributions in the vertical direction, which is called Landau–Levich regime.^[^ ^107^ ^]^ On the contrary, high coating speed will lead to thick film in this regime. Moreover, polymer aggregation and phase‐separation behaviors are closely related to fluid flow during the coating process, including mass transport of solute in solution through convection and diffusion, and mass transport of solvent during evaporation. In addition, during the evaporation process, the solvent evaporation rate at the periphery is higher than that in the center. To compensate the solvent loss at the periphery, an outward capillary flow will be generated. A coffee‐ring stain is caused since the solute is transported outward by the capillary flow, leading to the formation of pinholes and inhomogeneity. The main reason for the non‐ideal morphology is the mismatch of the aforementioned processes on the time scale.^[^ 108, 109 ^]^ Therefore, it is necessary to regulate the fluid mechanics for molecular crystallization and phase‐separation behavior. Ma et al.^[^ ^110^ ^]^ designed patterned blade micro‐cylinder arrays by photolithography to maximize the extensional and shear strain rates of fluids (**Figure** **10**a–c). Owing to the synergistic effect of extensional and shear flow, PM6 polymer chains show tighter packing and arrangement in the subsequent evaporation process, while Y6 molecules are sufficiently crystallized. Furthermore, the patterned blade can promote the orderly alignment of molecules and the enhancement of crystallinity to drive an optimized phase separation. Thus, the patterned blade‐coated PM6:Y6 films yield a PCE of 15.93% as compared to 14.55% from a normal blade. Li et al.^[^ ^111^ ^]^ developed a reversible and sequential layer‐by‐layer (RS‐LBL) deposition method with sequential twice forward/reverse blade‐coating of polymer donor and forward blade‐coating of Y6 acceptor to precisely control fluid mechanics of PM6:Y6 active layer (Figure 10d). They found that the non‐Newtonian fluid feature of PM6 primarily dominates the wedge‐shaped mass/phase distribution in the active layer, along with the gradually reduced film thickness in the blade‐coating direction. Moreover, the heterogeneous phase aggregation and crystallization in the large‐area active layer result in unbalanced hole/electron mobility and increased charge recombination losses in sub‐cells, leading to poor efficiency in the solar modules. Through using the RS‐LBL strategy, uniform morphology and favorable phase separation and crystallization are obtained in the 10 × 10 cm^2^ active layer. As a result, the RS‐LBL‐based OSCs show excellent operational stability, and an outstanding PCE of 13.47% is achieved with significantly suppressed charge recombination losses in the 36 cm^2^ large‐area OSC module.

## 6 FTEs

Large‐area flexible OSCs require immediate development to realize their practical application of flexible and portable power sources. Currently, an FTE is one of the key factors affecting the photovoltaic and mechanical properties of large‐area flexible OSCs. The high‐performance FTEs should possess high optical transparency, good mechanical bending durability, low surface roughness, low sheet resistance, superior thermal expansion stability, and solution processability to be compatible with R2R process. In the following, we will review the current status of flexible OSCs with different flexible FTEs based on conventional ITO, metal grids, and metal nanowires. The performance of corresponding flexible OSCs devices are summarized in **Table** **6**.

**Table 6:** The performance of flexible OSCs devices

FTE	Sheet resistance [Ω sq^−1^]	Transmittance [%]	Device structure	Active layer	Area [cm^2^]	PCE [%]	References
PET/ITO	47.4	83.64	Conventional	P3HT:PC_61_BM	N/A	1.88	[112]
PET/ITO	N/A	N/A	Inverted	SMD2:ITIC‐Th	80	5.25	[113]
PET/Ag grid:PH1000/PH1000	4.5	N/A	Conventional	P3HT:PCBB‐C8	1.21	1.36	[114]
PET/Ag mesh/PH1000:AgNWs‐20	6.0	86	Inverted	PM6:IT‐4 F	0.1	12.07	[6]
PET/Ag grid/AgNWs:PEI–Zn	20	N/A	Inverted	PM6:Y6:PC_71_BM	54	13.2	[115]
PET/Em‐Ag/AgNWs:AZO‐SG	18	95 (excluding the substrate)	Inverted	PM6:Y6	0.0737	15.21	[5]
PET/Em‐Ag/AgNWs‐IL	11.5	95 (excluding the substrate)	Inverted	PM6:BTP‐eC9:PC_71_BM	1.0	15.82	[39]

### 6.1 ITO‐Based FTEs

ITO has been widely used in electronic and optoelectronic devices as transparent electrodes due to its low sheet resistance (10–15 Ω sq^−1^) and high transmittance (>90%). However, the thermal‐radiation‐generated ITO layer by sputtering can easily damage the flexible substrate. Even worse, ITO is rather brittle and often cracks when bent. Despite these issues, ITO is still widely used to fabricate flexible OSCs due to the scarcity of commercial FTE materials. Kim et al.^[^ ^112^ ^]^ reported PET/ITO electrodes via continuous room‐temperature sputtering in an R2R sputtering system. They found that the adhesion between PET substrate and ITO was enhanced by treating PET surface with argon‐ion beam, thus obtaining ITO electrode with good mechanical durability and robustness. As a result, the PET/ITO electrodes showed their high sheet resistance of 47.4 Ω sq^−1^ with a transmittance of 83.46%, leading to a PCE of 1.88% in P3HT:PC_61_BM‐based spin‐coated flexible OSCs. Moon et al.^[^ ^113^ ^]^ designed a new random building block donor terpolymer SMD2 to manufacture efficient and high‐performance flexible OSC modules via slot‐die coating. By developing a donor polymer SMD2, which allows the utilization of a post‐treatment‐free process, a maximum PCE of 5.25% (80 cm^2^) was achieved owing to the balanced miscibility and crystallinity. Although the PET/ITO flexible electrode has been successfully applied in large‐area flexible OSCs, the brittle nature of the ITO layer is a critical drawback for its application. Therefore, it is important to explore novel FTEs materials for the future application of the flexible OSCs.

### 6.2 Metal‐Grids‐Based FTEs

**Figure 11:**
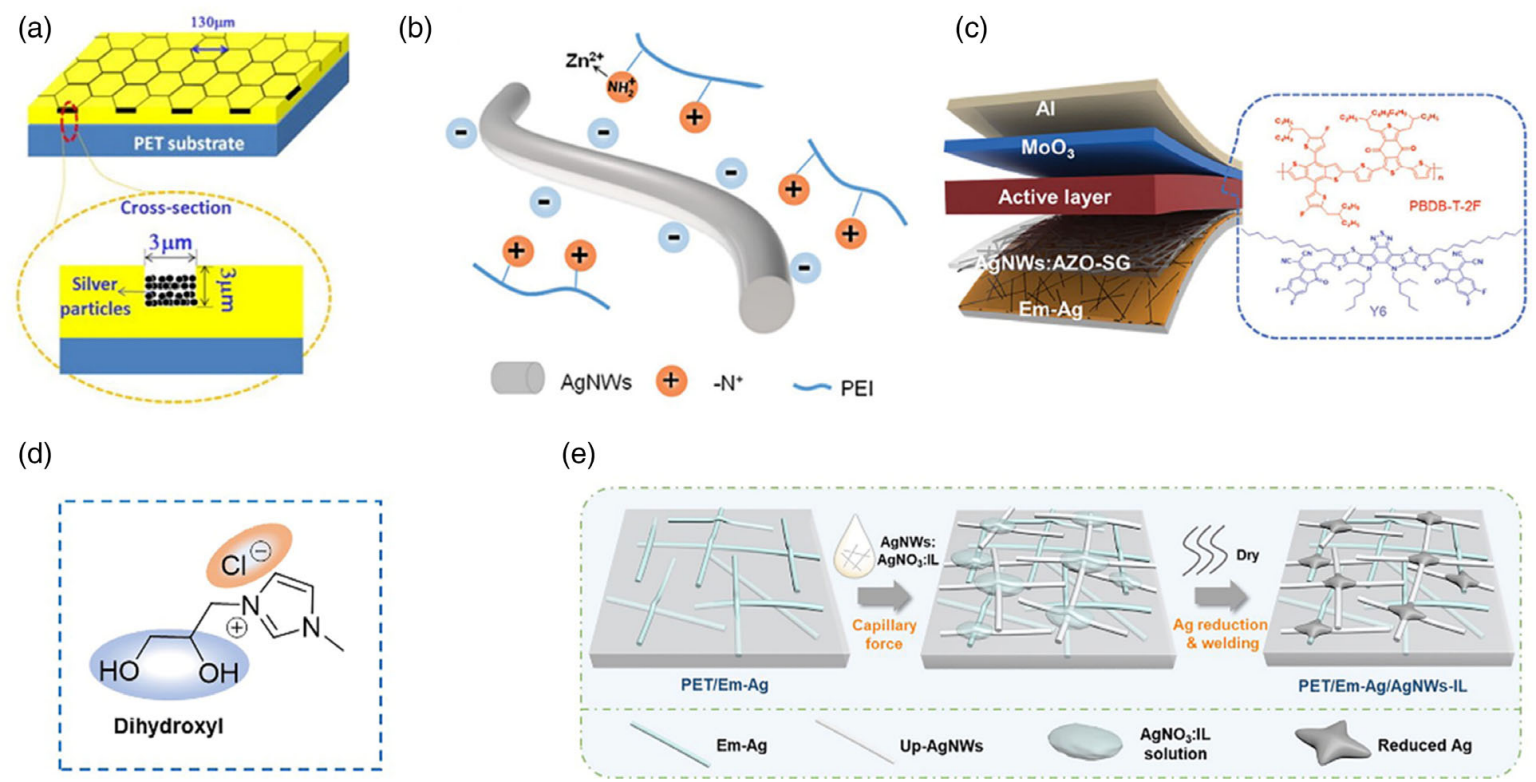
a) Schematic illustration of high‐resolution Ag‐grid embedded in PET substrate. The black line represents the Ag‐grid, the yellow areas are pastern, and the blue areas are PET substrate. Reproduced with permission.^[^ ^114^ ^]^ Copyright 2013, Elsevier. b) Schematic illustration of electrostatic interaction between positively charged amine and negatively charged AgNWs surface. Reproduced with permission.^[^ ^115^ ^]^ Copyright 2021, Wiley‐VCH. c) Schematic illustration of a flexible OSCs and molecular structures of the donor PBDB‐T‐2F and the acceptor Y6 in the active layer. Reproduced with permission.^[^ ^5^ ^]^ Copyright 2020, Wiley‐VCH. d) Chemical structure of the ionic liquid. e) Schematic diagram of the Ag reduction and welding processes involved in the fabrication of AgNW FTEs. d,e) Reproduced with permission.^[^ ^39^ ^]^ Copyright 2022, American Chemical Society.

Metal grids, especially Ag grids, have been widely used in many inorganic and flexible organic electronics, where the metal fingers provide patterned paths for conducting electricity and the gaps between the figures are the light windows for transmitting light. The electrical and optical properties of metal grids can be optimized by tuning the grid parameters, including aspect ratio, line width, depth, and pitch size. However, the surface roughness and thickness of the grids, especially for OSCs with a several hundred nanometers photoactive layer, should be taken seriously during fabrication. Chen et al.^[^ ^114^ ^]^ patterned a high‐resolution hexagonal Ag grid (*W* = 3 μm) on PET substrate (**Figure** **11**a), which showed low sheet resistance (0.5 Ω sq^−1^) and high transmittance (85%). Subsequently, they developed a new transparent electrode PET/Ag grid:PH1000/PH1000 (sheet resistance, 4.5 Ω sq^−1^) to improve surface flatness and wettability of the PET/Ag grid. The PH1000 mixed within Ag grids partially fills the pores and thus enhances the conductivity. Consequently, the spin‐coated large‐area (1.21 cm^2^) flexible OSCs based on the optimal electrode showed a PCE of 1.36%. Later, Li et al.^[^ ^6^ ^]^ fabricated flexible composite electrode PET/Ag mesh/PH1000:AgNWs‐20 by doping 20 wt% AgNWs into PH1000. The flexible composite electrode showed a sheet resistance as low as 6.0 Ω sq^−1^ and high transmittance of 86% at 550 nm wavelength. More importantly, its surface roughness can be reduced to 8.62 nm. The spin‐coated flexible OSCs based on this electrode achieved a record PCE of 12.07% with high reproducibility. Recently, Zhou et al.^[^ ^115^ ^]^ reported a novel flexible composite electrode PET/Ag grid/AgNWs: zinc‐chelated polyethyleneimine (PEI–Zn, Figure 11b). After filled with PEI–Zn, the transmittance of Ag grid/AgNWs:PEI–Zn became slightly higher than that of AgNWs, and the electrode surface was more smooth (2.3 nm). This might be attributed to the polymer matrix PEI–Zn that can well wrap the AgNWs and act as an antireflection layer. As a result, the spin‐coated flexible module with an area of 54 cm^2^ exhibits a PCE of 13.2%. At present, the Ag grids have been demonstrated in flexible OSCs because of their superior mechanical durability. However, the complexity and high cost of preparing Ag grids are problems that need to be solved in the future.

### 6.3 Metal‐Nanowires‐Based FTEs

Metal nanowires, particularly AgNWs, exhibit great potential as effective highly conductive FTE due to their high transmittance, low sheet resistance, and good mechanical flexibility. Furthermore, the properties of AgNWs allow them to be coated on arbitrary substrates via solution‐based processing, which is beneficial for large‐area production. However, AgNWs films still suffer from several shortcomings, such as low coverage, random distribution, and low adhesion. Li et al.^[^ ^5^ ^]^ used a “welding” strategy to design an FTE with tight binding of the upper electrode and the underlying substrate (Figure 11c). Owing to the capillary force effect and secondary growth of Al‐doped ZnO (AZO) from solution, the AgNWs parasitic absorption from electrode compositions can be effectively avoided and the unfavorable AgNW junction sites can be successfully welded. Furthermore, they embed AgNWs in the UV‐curing resin, thus enabling linkage between the AgNWs in the upper hybrid electrode and the underlying substrate. The resultant welding AgNW‐based FTE exhibits a low sheet resistance of 18 Ω sq^−1^ and a high transmittance of 95% at 550 nm. They also fabricated a chemically welded AgNWs FTE on a PET/Em‐Ag substrate by coating an AgNW solution containing a low concentration of AgNO_3_ and a reduction agent ionic liquid (IL, Figure 11d).^[^ ^39^ ^]^ The Cl^−^ anions in the IL could regulate the Ag^+^ concentration through the formation and dissolution of AgCl, and the dihydroxyl group in the IL reduces the slowly released Ag^+^ to form the metal Ag. The reduced Ag grew in situ in a twin‐crystal mode, tightly welding the junction sites of the AgNWs and realizing an atomic‐level contact between them, which helps to decrease the sheet resistance (11.5 Ω sq^−1^) and enhance the mechanical stability of the FTEs (Figure 11e). As a result, the spin‐coated flexible OSCs based on this chemically welded FTE achieved record PCEs of 17.52% (0.062 cm^2^) and 15.82% (1.0 cm^2^). Although the flexible FTEs based on the AgNWs exhibit some advantages such as good flexibility and high conductivity, the synthesis of high‐quality AgNWs and the stability issue of AgNWs should be addressed.

### 6.4 Transparent Top Electrode

At present, in the process of preparing OSCs, some interface layers (MoO_3_) and metal electrodes need to be prepared by evaporation. However, the cost of vacuum equipment and its follow‐up maintenance are relatively high, which is limited in mass production. One of the effective ways to realize the batch production of OSCs is to adopt the method of the all‐solution fabrication process to realize the high‐performance OSCs.^[^ 116, 117, 118 ^]^ AgNWs is a commonly used electrode material for solution‐processing top electrode due to its good solution processability and photoelectric performance. Ade et al.^[^ ^119^ ^]^ proposed a bimodal AgNW (AgNW‐BM) electrode comprised AgNWs of two different aspect ratios as the top electrode. The AgNW top electrode was deliberately bimodal in size distribution and comprised two different aspect ratio AgNWs to suppress the agglomeration in the film‐formation process and ensure a good penetration network, leading to improved electrical conductivity and optical transmittance. Finally, the all solution prepared OSCs with PM6:Y6 as the active layer obtained a PCE of 9.79% with an average visible transmittance of 23%. In addition, the interface layer poly(3,4‐ethylenedioxythiophene):poly(styrene sulfonate) (PEDOT:PSS) is usually explored in all solution prepared OSCs that suffer the instability issue due to low wettability and acidity. To overcome this issue, Zhou et al.^[^ ^120^ ^]^ reported an alcohol‐dispersed formulation (PEDOT:F) by employing perfluorinated sulfonic acid ionomers as counterions to replace the PSS counterion. The ionomer can be dispersed in water or alcohol, leading to PEDOT:F dispersion that can be prepared in alcohol. More importantly, because of the small surface tension of the alcohol solvent and the small ionization constant of the acid in the alcohol, the alcohol‐dispersed formulation has good wettability and low acidity, avoiding the shortcomings of aqueous PEDOT:PSS. As a result, all solution‐prepared OSC module based on PEDOT:F were obtained with a PCE of 13.07% (12.2 cm^2^) and an excellent operational stability. Furthermore, reducing the surface roughness of the top electrode and enhancing the Ohmic contact with the interface layer are urgent topics for all solution printing.

## 7 Stability for Large‐Area OSCs

To realize the commercialization of OSCs, device stability issues cannot be ignored. It is generally accepted that the operating lifetime of OSC modules for commercial applications should be at least 10 years.^[^ 121, 122 ^]^ However, the morphology of the organic active layer is a kinetically trapped state and thus not permanent. When the OSCs are exposed to device operating conditions (humidity, oxygen, and heat), organic molecules in the active layer will diffuse slowly or recrystallize over time until reaching a thermodynamic equilibrium state.^[^ ^123^ ^]^ Therefore, it is a major subject to study the stability of device structure and photovoltaic materials under various environments. In this section, we will mainly discuss the recent advances in the stability of large‐area OSCs.

The intrinsic molecular structure of organic active layer materials may be one of the most important factors that affect the devices stability. Tuning the crystallinity of donor materials is considered to be an effective approach for enhancing device stability. Durrant et al.^[^ ^124^ ^]^ reported that the photochemical stability of donor polymers is greatly affected by their crystallinity which is related to the oxygen‐quenching efficiency and lifetime of polymer triplet states. They showed that over 80% of triplet excitons are quenched by oxygen in the most amorphous polymers (RRa–P3HT or GeIDT–BT). However, most crystalline polymers such as DPPTT‐T showed negligible oxygen‐quenching affection due to the less exposure time to interact with oxygen in comparison with amorphous polymers. Park et al.^[^ ^125^ ^]^ found that photooxidation reactions are generally favored at electron‐rich sites in organic molecules. They demonstrated that electron‐donating solubilizing groups should maintain a separation distance from the electron‐rich photoreactive sites. As a result, the copolymer of diketopyrrolopyrrole (DPP) and dithieno[3,2‐b:2′,3′‐d]thiophene (DTT) (PDPPDTT) with the low‐electron‐donating abilities of dithienothiophene and alkyl‐chain showed a high photochemical stability, which led to a significantly enhanced device stability. In addition, developing morphology stability acceptor materials with a high glass‐transition temperature (*T*_g_) is also critical to improve the stability of large‐area OSCs. Min et al.^[^ ^126^ ^]^ introduced a novel NFA 2,2′‐([4,9‐bis(1‐hexylheptyl)‐4,9‐dihydrothieno[3,2‐b]thieno[2′,3′:4,5]pyrrolo[2,3‐f]indole‐2,7‐diyl]bis{(Z)methylylidene[(2Z)‐3‐oxo‐1H‐indene‐2,1 diylidene]})dimalononitrile (TPIIC) as the third component into PM6:Y6 host devices, effectively improving the molecular crystallinity and *T*_g_ in the blend film. The PM6:Y6:TPIIC ternary devices retained 74.7% and 88.0% of their initial efficiency, after up to 1000 h of the light‐soaking and thermal stress treatments (85 °C), respectively. In contrast, the PM6:Y6 host system exhibited worse photostability and thermal stability, probably due to its unstable molecular configuration and low *T*_g_ of Y6. In addition, selecting appropriate additives is also an effective way to increase *T*_g_. Chen et al.^[^ ^127^ ^]^ found that 1,4‐butanedithiol solvent additive significantly improved thermal stability of PTB7‐Th:PC_71_BM system, in contrast to the traditional DIO additive. The significantly improved thermal stability after processing with 1,4‐butanedithiol is mainly contributing to optimal distribution of fullerenes in donor matrix, leading to enhanced *T*_g_ of polymer. Furthermore, the selection of low‐boiling‐point 1,4‐butanedithiol could effectively avoid the residue of high‐boiling‐point solvent additive like DIO, leading to higher *T*_g_ for polymer and significantly improved thermal stability.

In addition, various encapsulation techniques have been developed to greatly resist moisture and UV illumination. Kim et al.^[^ ^128^ ^]^ reported a novel UV‐cut filter on the front of glass substrates to successfully block most UV photons below 403 nm, leading to significantly enhanced UV illumination stability with maintained 90% efficiency after 180 min UV aging. Venkataraman et al.^[^ ^129^ ^]^ developed a self‐healing polymer sealant polyisobutylene to prevent moisture erosion and gave 85% of its initial PCE even after 20 days in the air, while the PCE of unencapsulated one decreased rapidly within 2 days.

## 8 Conclusions and Outlook

To date, the certificated PCEs of small‐area single‐junction OSCs have reached over 19%, representing a potentially significant step in the path toward commercialization. Unfortunately, the coating methods and materials used for small‐area OSCs are not completely suitable for large‐area manufacturing. In this review article, we summarized the latest development of large‐area OSCs, including the demand for the active layer and interface layer materials, coating techniques, morphology regulations, novel FTEs, and stability research. Developing appropriate coating techniques is an important part of large‐area device production, including blade‐coating, slot‐die coating, gravure printing, and inkjet printing. In addition, the design of active layer and interface materials with properties suitable for large‐area fabrication also is essential to improve device performance, such as favorable thickness insensitivity, predominate face‐on stacking orientation, good solubility, and crystallinity. Obtaining uniform nanoscale phase‐separation morphology in the photoactive layer is also the key to achieving high‐performance large‐area OSCs. At present, solvent engineering, additive engineering, coating speed/temperature, LBL‐processing method, and fluid mechanics controlling are the main means to regulate the morphology. High‐conductivity transparent electrodes are important factors for the large‐area flexible OSCs concerning the practical application of flexible and portable power sources. Finally, the stability of OSCs devices is the final step in commercialization. Based on the aforementioned discussions, several approaches are proposed for future research on large‐area OSCs: 1) Developing active layer materials with high thickness insensitivity. Although many efficient thick‐film devices have been developed, few have been reported over 500 nm. It urgent needed to design the highly crystalline donors and acceptors via rational design strategies, including the introduction of fluorine/chlorine atoms on the polymer backbone, side‐chain engineering, halogenation on the end groups, etc. 2) A comprehensive understanding of the fluid mechanics is necessary toward their application in large‐area OSCs. Compared with small molecules, the polymers usually exhibit non‐Newtonian (shear‐rate‐dependent) behaviors like shear thinning, shear thickening, or even (time‐dependent) thixotropy, which may have deep implications for the control of film morphology. Therefore, it is important to deeply understand the fluid flow behavior with the help of some simulation tools, such as finite‐element analysis, COMSOL Multiphysics, and computational fluid dynamics. Subsequently, devising ways is needed to control the flow behavior without sacrificing the film Quality. 3) It is critical to understand film‐formation mechanisms based on scalable coating methods for guiding process optimization. The film formation and drying behaviors are different by using different coating or printing methods in large‐area devices. And the process of film formation during coating can be well monitored by in situ characterization (in situ UV–visible absorption, grazing‐incidence wide‐angle X‐ray scattering, etc.). 4) Developing high‐quality FTEs with ultralow sheet resistance and smooth surface for reducing series resistance of the large‐area flexible OSCs is also essential. The high‐quality and low‐cost FTE needs to be further developed especially for composite electrodes as they can combine their respective advantages. 5) It is significant to optimize the large‐area module layout (including P1–P2–P3 interconnection area) as well as the high‐resolution short‐pulse laser‐structuring processes involved in the manufacturing of such efficient OSCs modules. The length and the width of the single cell have a great influence on the internal resistance, thereby affecting the electrical losses in the device. Moreover, the length of the single cell and module connection region determine the geometric fill factor (GFF) of the modules, which determines the geometric losses. For example, a too‐narrow P2 line would reduce the connection between the top electrode and the bottom electrode, which may increase interconnect resistance, while a too‐wide P2 line would cause GFF losses. To break the limitation of patterning lengths and improve the GFF of the modules, laser patterning which employs strong irradiations from a focused laser beam to remove materials from a surface is adopted as a pattern assistant technique. However, the wavelength and pulse duration of patterning parameters should be properly optimized to control the absorption of the laser radiation in the processed materials and the extent of heat influence on the surrounding materials. Femtosecond laser pulses allow very precise and pure laser processing of solids without collateral damage to the surrounding layers and layers lying beneath is needed to be applied for the high‐performance OSC modules; and 6) low‐cost and high‐quality active layer materials also are urgent to be developed due to the future R2R high‐throughput technology requiring a large number of materials. In addition, the development of effective encapsulation materials and processes can ensure efficient and stable large‐area OSCs also need urgent attention. If these challenges can be addressed properly, OSCs may enter the market in the near future.

## Conflict of Interest

The authors declare no conflict of interest.
